# Control of immediate early gene expression by CPEB4-repressor complex-mediated mRNA degradation

**DOI:** 10.1186/s13059-022-02760-5

**Published:** 2022-09-12

**Authors:** Fabian Poetz, Svetlana Lebedeva, Johanna Schott, Doris Lindner, Uwe Ohler, Georg Stoecklin

**Affiliations:** 1 Division of Biochemistry, Mannheim Institute for Innate Immunoscience (MI3) and Mannheim Cancer Center (MCC), Medical Faculty Mannheim, Heidelberg University, Ludolf-Krehl-Str. 13-17, 68167 Mannheim, Germany; 2 Center for Molecular Biology of Heidelberg University (ZMBH), 69120 Heidelberg, Germany; 3 Berlin Institute for Molecular Systems Biology (BIMSB), Max Delbrück Center for Molecular Medicine, 10115 Berlin, Germany; 4 Department of Biology, Humboldt Universität Berlin, 10099 Berlin, Germany

**Keywords:** CCR4-NOT complex, Deadenylation, mRNA turnover, Acetylation, CPEB4, Immediate early genes, Cytoplasmic polyadenylation element

## Abstract

**Background:**

Cytoplasmic polyadenylation element-binding protein 4 (CPEB4) is known to associate with cytoplasmic polyadenylation elements (CPEs) located in the 3′ untranslated region (UTR) of specific mRNAs and assemble an activator complex promoting the translation of target mRNAs through cytoplasmic polyadenylation.

**Results:**

Here, we find that CPEB4 is part of an alternative repressor complex that mediates mRNA degradation by associating with the evolutionarily conserved CCR4-NOT deadenylase complex. We identify human CPEB4 as an RNA-binding protein (RBP) with enhanced association to poly(A) RNA upon inhibition of class I histone deacetylases (HDACs), a condition known to cause widespread degradation of poly(A)-containing mRNA. Photoactivatable ribonucleoside-enhanced crosslinking and immunoprecipitation (PAR-CLIP) analysis using endogenously tagged CPEB4 in HeLa cells reveals that CPEB4 preferentially binds to the 3′UTR of immediate early gene mRNAs, at G-containing variants of the canonical U- and A-rich CPE located in close proximity to poly(A) sites. By transcriptome-wide mRNA decay measurements, we find that the strength of CPEB4 binding correlates with short mRNA half-lives and that loss of CPEB4 expression leads to the stabilization of immediate early gene mRNAs. Akin to CPEB4, we demonstrate that CPEB1 and CPEB2 also confer mRNA instability by recruitment of the CCR4-NOT complex.

**Conclusions:**

While CPEB4 was previously known for its ability to stimulate cytoplasmic polyadenylation, our findings establish an additional function for CPEB4 as the RNA adaptor of a repressor complex that enhances the degradation of short-lived immediate early gene mRNAs.

**Supplementary Information:**

The online version contains supplementary material available at 10.1186/s13059-022-02760-5.

## Background

Transcription and mRNA turnover jointly determine the steady-state level and expression dynamics of any given mRNA. Like in other eukaryotes, mammalian mRNAs vary greatly in their stability ranging from minutes to several hours [1–4]. The regulation of mRNA stability is a critical step in the control of gene expression and represents an efficient way of adjusting the transcriptome to changing requirements. Depending on the cellular system, mRNA turnover has been estimated to account for approximately 15–50% of changes in gene expression in response to extrinsic stimuli [5–7]. Moreover, mRNAs encoding proteins with similar functional roles undergo simultaneous decay, thus forming post-transcriptional regulons ensuring their coordinate expression [8, 9].

The poly(A) tail acts as an integral determinant of both mRNA stability and translation. In eukaryotes, degradation of most mRNAs is initiated by deadenylation and relies on the evolutionarily conserved carbon catabolite repression 4—negative on TATA-less (CCR4-NOT) complex, which acts as the major deadenylase from yeast to humans [10–13]. CCR4-NOT-mediated shortening of poly(A) tails is catalyzed by two exoribonucleases, CCR4 and CCR4-associated factor 1 (CAF1). Whereas CCR4 activity depends on the presence of poly(A)-binding protein (PABP), CAF1 deadenylates poly(A) tails that are not bound by PABP [14, 15]. CCR4-NOT-mediated deadenylation ultimately leads to deprotection of the mRNA 3′end, leading to further degradation of the mRNA body from the 5′ or 3′end [16, 17]. The speed of translation elongation has a strong impact on mRNA stability as sub-optimal codons promote the degradation of mRNAs [18, 19]. Hence, protein synthesis and mRNA decay are highly interconnected processes where the CCR4-NOT complex fulfils the function of a coupling factor that monitors codon optimality within translating ribosomes [20].

Gene-specific changes in mRNA stability are mediated by binding of destabilizing RNA-binding proteins (RBPs) to distinct sequence motifs that are typically located in mRNA 3′UTRs. Apart from members of the BTG/TOB family, which act as general activators of mRNA decay through direct interaction with the deadenylase CAF1 [21–24], a multitude of decay-promoting RBPs induces selective degradation of individual transcripts via direct recruitment of the CCR4-NOT complex. Among these are the AU-rich element (ARE)-binding protein ZFP36/Tristetraprolin [25–27], the miRNA-mediated silencing complex (miRISC) scaffolding subunit GW182/TNRC6 [28, 29], the RNA stem-loop binding protein RC3H1/Roquin [30, 31], members of the Nanos/Pumilio family [32–34], and the m^6^A reader protein YTHDF2 [35].

The poly(A) tail length of mRNAs can further be regulated in the cytoplasm by members of the cytoplasmic polyadenylation-binding (CPEB) protein family, which facilitate cytoplasmic polyadenylation and translational activation during meiotic and mitotic cell divisions [36–38]. Notably, CPEBs can act as molecular switches that trigger, depending on their phosphorylation status, either the lengthening of poly(A) tails as part of an activator complex, or the shortening of poly(A) tails as part of a repressor complex [39]. Among the four mammalian CPEB proteins, CPEB4 was found to have a pro-tumorigenic function promoting the expression of key oncogenic drivers [40, 41]. Similar to the other CPEBs, CPEB4 is known to associate via its two conserved tandem RNA recognition motifs (RRMs) with the cytoplasmic polyadenylation element (CPE), a U-rich and A-rich motif located in the 3′UTR of specific mRNAs [42, 43]. In most systems studied so far, CPEB4 maintains poly(A) tail length and enhances the translation of individual mRNAs [40, 41, 44]. Two other CPEB family members, CPEB1 and CPEB3, were found to initiate the degradation of specific mRNAs through indirect recruitment of CAF1 via TOB proteins [45, 46]. However, the role of CPEB4 in mRNA deadenylation and decay remains poorly investigated.

Protein acetylation represents a prevalent post-translational modification of RNA-associated proteins [47, 48], which impacts post-transcriptional regulatory networks by modulating their interaction with RNA [49–52] or their localization [53–56]. Previously, we found that inhibition of histone deacetylase 1 (HDAC1) and 2 induces widespread degradation of normally stable poly(A)-containing RNAs, a process that depends on the CCR4-NOT complex [57] and is regulated by its stably associated inhibitor RNF219 [58]. Here, we set out to identify RBPs involved in the regulation of mRNA turnover following an increase in cellular acetylation and describe CPEB4 as an RBP that is induced by acetylation and triggers the degradation of immediate early gene (IEG) mRNAs via recruitment of the CCR4-NOT complex.

## Results

### Poly(A) RNA interactome capture identifies changes in the mRNA-bound proteome upon HDAC inhibition

**Fig. 1:**
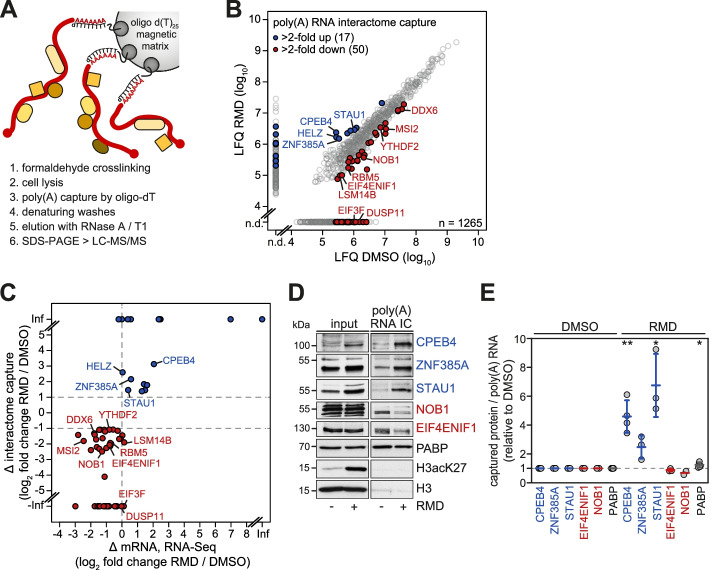
Poly(A) RNA interactome capture upon HDAC inhibition by RMD. **A** Schematic illustrating the poly(A) RNA interactome capture procedure. Cells were chemically crosslinked with 0.05% formaldehyde, and after cryogenic lysis, poly(A) RNA was affinity purified using an oligo d(T)_25_ magnetic matrix. Following denaturing washes, bound proteins were eluted and subjected to LC-MS/MS. **B** HeLa cells were treated with the class I-specific HDAC inhibitor romidepsin (RMD, 20 nM) or an equal volume of solvent (DMSO) for 16 h prior to comparative poly(A) RNA interactome capture (*n* = 2). The scatter plot depicts log_10_-transformed label-free quantification (LFQ) values from the ensuing LC-MS/MS proteomics analysis; only proteins enriched >10-fold over non-crosslinked controls are depicted. Candidate proteins displaying an at least 2-fold increased (blue) or decreased (red) association with poly(A) RNA upon RMD treatment in two biological repeats are highlighted; n.d.: not detected. **C** Scatter plot depicting the fold-change in poly(A) RNA association in relation to the fold-change in mRNA abundance as determined by RNA-Seq. Only candidate proteins with increased (blue) or decreased (red) binding to poly(A) RNA upon RMD treatment are shown; Inf, infinite. **D** Validation of selected candidate proteins by western blot analysis after poly(A) RNA interactome capture. **E** Differential association of selected candidate proteins from **D** was quantified after normalizing to the amount of co-purified poly(A) RNA (Additional file 2: Fig. S1A). Data are presented as mean ± SD (*n* ≥ 2), indicated *p*-values were calculated using a two-sided, one-sample *t*-test; * *p* < 0.05, ** *p* < 0.01

To explore acetylation-dependent changes in the poly(A) RNA-bound proteome, we made use of poly(A) RNA interactome capture (IC), a crosslinking-based approach by which poly(A) RNA-associated proteins are isolated upon hybridization to oligo-dT [59, 60]. While the original protocol relies on UV crosslinking, we established a revised protocol for poly(A) RNA IC that requires less cellular material due to more efficient chemical crosslinking with formaldehyde, similar to approaches used in several recent studies [61–63]. Formaldehyde-assisted crosslinking was followed by cryogenic lysis and affinity purification of poly(A) RNA using oligo(dT)_25_ magnetic beads. After removal of non-covalently bound proteins during denaturing washes, the mRNA-bound proteome was eluted and subsequently analyzed by liquid chromatography tandem mass spectrometry (LC-MS/MS, Fig. 1A).

By applying this approach to HeLa cells, we were able to identify 664 MaxQuant-assigned protein groups in two independent biological replicates in the control condition (DMSO, Additional file 1). These groups correspond to 701 genes, whose proteins showed an at least 10-fold enrichment over the non-crosslinked control. Quality assessment confirmed that poly(A) RNA IC does not compromise poly(A) RNA integrity (Additional file 2: Fig. S1A). Moreover, the proteins we identified are in good agreement with previously published RNA IC datasets from HeLa [59] and human embryonic kidney 293 (HEK293) cells [64] (Additional file 2: Fig. S1B) and showed a similar distribution of RBP classes (Additional file 2: Fig. S1C). Hence, formaldehyde-assisted crosslinking in conjunction with poly(A) RNA IC enables reliable RBP identification from 35–40-fold lower amounts of cellular material as compared to the original protocol using UV crosslinking [59, 60].

To analyze the effect of HDAC inhibition on the composition of the poly(A) RNA-bound RBPs, we used the class I-specific HDAC inhibitor Romidepsin (RMD) [65], which we previously found to potently induce poly(A) RNA degradation at nanomolar concentrations [57]. Poly(A) RNA IC from HeLa cells treated with 20 nM RMD for 16 h revealed 577 protein groups corresponding to 615 genes (Additional file 3). By requesting a fold-change of more than two in two independent biological replicates, RMD treatment was found to cause enrichment and disenrichment of 17 and 50 candidate RBPs, respectively (Fig. 1B). Interestingly, differential association of candidate RBPs to poly(A) RNA correlates well with differential abundance of the corresponding mRNAs (Fig. 1C, Additional files 4 and 5), indicating that changes in gene expression are the major driver of RMD-induced alterations in the poly(A) RNA-bound proteome. Validation of selected candidate proteins by western blotting confirmed enrichment of CPEB4, zinc finger protein 385A (ZNF385A), and STAU1, whereas EIF4ENIF1 (also known as 4E-T) and NOB1 were disenriched among poly(A) RNA-bound proteins upon RMD treatment (Fig. 1D, E).

### CPEB4 is selectively upregulated upon HDAC inhibition

The two CPEB family members CPEB1 and CPEB3 are known to induce the degradation of selected mRNAs by recruitment of the CCR4-NOT complex-associated deadenylase CAF1 [45, 46]. Given the importance of the CCR4-NOT complex in acetylation-induced mRNA turnover [57], we decided to focus on CPEB4, whose expression and binding to mRNA is strongly enhanced by RMD (Fig. 1C–E).

**Fig. 2:**
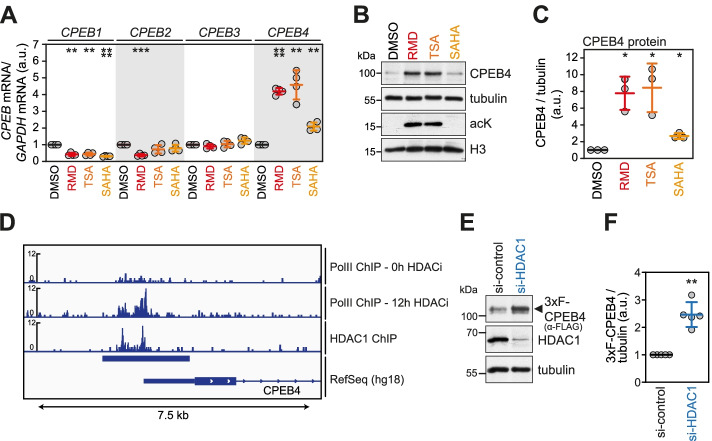
Upregulation of CPEB4 expression by HDAC inhibition. **A** *CPEB1-4* mRNA levels were measured by qRT-PCR in HeLa cells treated with 20 nM RMD, 500 nM Trichostatin A (TSA), 500 nM suberoylanilide hydroxamic acid (SAHA), or an equal volume of solvent (DMSO) for 16 h. GAPDH mRNA was used for normalization; data are presented as mean ± SD (*n* = 4). **B** Western blot analysis of CPEB4 protein expression in HeLa cells after treatment with HDAC inhibitors as described in **A**. **C** Quantification of western blots as in **B**; tubulin was used for normalization; data are presented as mean ± SD (*n* = 3). **D** Integrative Genomics Viewer tracks of RNA polymerase II (PolII) and HDAC1 occupancy; ChIP-Seq data are derived from CD4^+^ T-cells following HDAC inhibition (HDACi) with 100 ng/ml TSA and 2 mM Sodium Butyrate for 12 h [67]. **E** Western blot analysis of endogenously tagged 3xFLAG (3xF)-CPEB4 protein expression in HeLa cells following transfection with either control or HDAC1-targeting siRNAs for 48 h. **F** Quantification of western blots as in **E**; tubulin was used for normalization; data are presented as mean ± SD (*n* = 5). *p*-values in **A**, **C**, and **F** were calculated using a two-sided, one-sample *t*-test; * *p* < 0.05, ** *p* < 0.01, *** *p* < 0.001, **** *p* < 0.0001

First, we tested if CPEB4 expression is enhanced by HDAC inhibitors in general, using the pan-HDAC inhibitor Trichostatin A (TSA) and the structurally related suberoylanilide hydroxamic acid (SAHA), an FDA-approved HDAC inhibitor used for the treatment of refractory cutaneous T-cell lymphoma [66]. Treatment of HeLa cells with all three HDAC inhibitors increased the expression of both CPEB4 mRNA and protein (Fig. 2A–C). Induction was most prominent with RMD and TSA treatment, leading to an approximately 8-fold increase in CPEB4 protein expression (Fig. 2B, C). CPEB4 protein expression was also elevated by HDAC inhibition in two melanoma cell lines (Additional file 2: Fig. S2A), where CPEB4 was described to have a function in cancer progression [41]. Notably, mRNA expression of the other CPEBs was either reduced (*CPEB1, CPEB2*) or not affected (*CPEB3*) by HDAC inhibition (Fig. 2A). Since HDAC inhibition augmented not only CPEB4 mRNA but also pre-mRNA levels (Additional file 2: Fig. S2B), HDAC inhibition most likely acts by increasing transcription of *CPEB4.* This observation was corroborated by the fact that the time-dependent induction of *CPEB4* mRNA, which reached a maximum after 8 h of RMD treatment, was abolished by transcriptional shut-off with actinomycin D (actD, Additional file 2: Fig. S2C). To further investigate the involvement of HDACs in CPEB4 transcription, we made use of ChIP-Seq data from primary human CD4^+^ T-cells [67]. The analysis revealed RNA polymerase II (PolII) occupancy immediately upstream of the first exon of CPEB4, which increased following 12 h of HDAC inhibition (Fig. 2D). Notably, PolII occupancy coincides with an HDAC1 binding site within the same dataset (Fig. 2D), suggesting a function of HDAC1 in CPEB4 transcription. We therefore investigated the effect of siRNA-mediated knock-down (KD) of HDAC1 on CPEB4 expression. To improve the detection of CPEB4 and enable its specific purification for subsequent experiments, we generated a stable HeLa knock-in cell line expressing endogenously tagged CPEB4 by CRISPR/Cas9-mediated genome editing. A single-stranded donor oligonucleotide was used to integrate a 3xFLAG (3xF) coding sequence at the N-terminus of CPEB4 (Additional file 2: Fig. S3A). Characterization of the HeLa-3xF-CPEB4 clone #81 used for all subsequent experiments revealed that it contains an in-frame, monoallelic integration of the 3xFLAG sequence downstream of the ATG start codon of CPEB4 (Additional file 2: Fig. S3B, C), and displays time-dependent induction of CPEB4 expression upon RMD treatment (Additional file 2: Fig. S3D). KD of HDAC1 led to a significant increase in 3xF-CPEB4 protein levels (Fig. 2E, F), validating HDAC1 as a negative regulator of CPEB4 expression. Taken together, the expression analysis demonstrates that among the four CPEB family members, CPEB4 is selectively upregulated by treatment with different HDAC inhibitors, likely involving HDAC1-mediated transcriptional regulation.

### CPEB4 interacts with the CCR4-NOT complex via TOB1 and mediates mRNA degradation

**Fig. 3:**
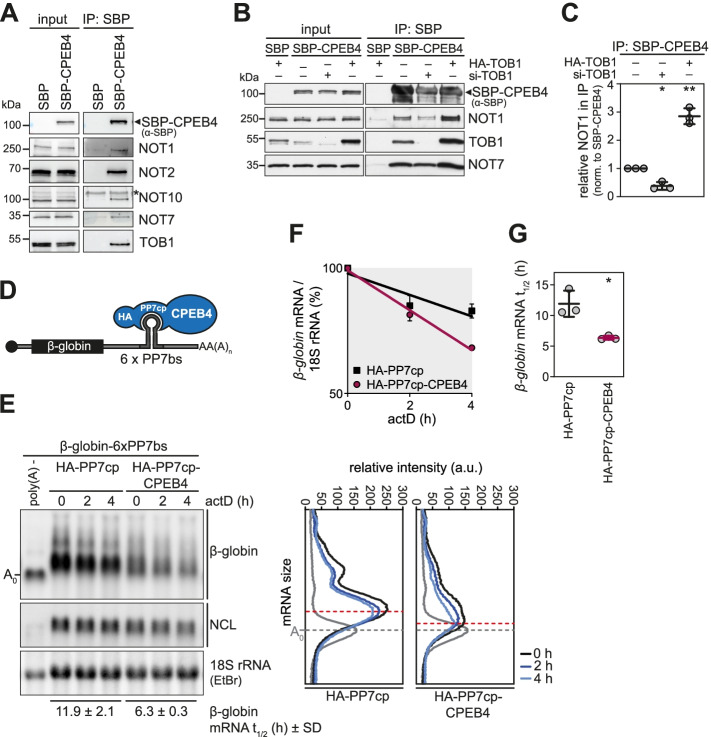
CPEB4-repressor complex formation by interaction with the CCR4-NOT complex. **A** Co-IP of endogenous NOT1, NOT2, NOT7, NOT10, and TOB1 with ectopically expressed SBP-CPEB4 in HeLa cells. IP was carried out using streptavidin beads; precipitated proteins were detected by western blot analysis using specific antibodies as indicated. The asterisk denotes a non-specific band. **B** Co-IP of endogenous NOT1, NOT7, and TOB1 with ectopically expressed SBP-CPEB4 in HeLa cells following treatment with 20 nM RMD. After siRNA-mediated KD of TOB1 or overexpression of HA-TOB1, the IP was carried out as in **A**. **C** The amount of co-precipitated NOT1 was quantified after normalization to the SBP-CPEB4 signal; data are presented as mean ± SD (*n* = 3). *p*-values were calculated using a two-sided, one-sample *t*-test. **D** Schematic illustration of the tethering assay. HA-CPEB4 fused to the bacteriophage PP7 coat protein (PP7cp) is co-expressed with a *β-globin* reporter mRNA containing 6 repeats of the PP7binding site (PP7bs). **E** For the tethering assay, HeLa cells were transiently transfected with HA-PP7cp or HA-PP7cp-CPEB4 together with a *β-globin* reporter mRNA containing 6 repeats of the PP7bs. Transcription was shut-off with 5 μg/ml actinomycin D (actD), and total RNA was extracted at indicated time points. Decay of the *β-globin* reporter mRNA was visualized by northern blot analysis; 18S rRNA was visualized by ethidium bromide (EtBr) staining after blotting; *nucleolin* (NCL) mRNA serves as additional loading control (left side). Deadenylation was visualized by densitometric analysis of the *β-globin* mRNA signal (right side). RNA digested with RNase H in presence of oligo-dT serves as a reference for fully deadenylated (poly(A)-) RNA. The signal intensity was plotted as a function of the poly(A) tail length and the maximum of the 4-h timepoint is indicated by a red dashed line. **F** The decay of *β-globin* mRNA is depicted after normalization to 18S rRNA (mean values ± SD; *n* = 3). **G** Half-lives of the *β-globin* mRNA in the tethering assays were calculated following first-order decay kinetics (mean values ± SD; *n* = 3). *p*-values were calculated using a two-sided, paired *t*-test; * *p* < 0.05, ** *p* < 0.01

CPEB1 and CPEB3 interact with the CCR4-NOT complex in a TOB1-dependent manner and thereby destabilize selected mRNAs such as *MYC* and *GLUR2* mRNA [45, 46]. Co-immunoprecipitation (co-IP) experiments demonstrate that ectopically expressed streptavidin binding peptide (SBP)-tagged CPEB4 associates with endogenous NOT1, NOT2, NOT7/CAF1a, NOT10 and the general mRNA decay factor TOB1 (Fig. 3A). Notably, the interaction between SBP-CPEB4 and NOT1 was found to depend on the abundance of TOB1 since KD of TOB1 significantly reduced the amount of co-precipitated NOT1, whereas overexpression of HA-TOB1 had the opposite effect (Fig. 3B, C). These results demonstrate that CPEB4 recruits the CCR4-NOT complex in a TOB1-dependent manner, which is in line with a previously proposed model for CPEB1- and CPEB3-mediated mRNA destabilization involving the formation of a CPEB-TOB-CAF1 ternary complex [45, 46].

We next investigated whether CPEB4 induces mRNA degradation using a tethering approach. A *β-globin* reporter mRNA harboring 6 repeats of the bacteriophage PP7 binding site (PP7bs) in its 3′UTR was co-expressed in HeLa cells with HA-CPEB4 fused to the PP7 coat protein (PP7cp), which binds tightly to the PP7bs [68] (Fig. 3D). Total RNA was extracted at regular time intervals following transcriptional shut-off with actD, and *β-globin* mRNA was visualized by northern blotting (Fig. 3E). Tethering of CPEB4 resulted in a two-fold acceleration of *β-globin* mRNA degradation as compared to tethering of HA-PP7cp alone (Fig. 3E–G). Moreover, densitometric analysis along the length of the reporter mRNA showed accelerated deadenylation upon CPEB4 tethering (Fig. 3E, Additional file 2: Fig. S4). Thus, CPEB4 forms a repressor complex by recruitment of the CCR4-NOT deadenylase and triggers the deadenylation and degradation of bound mRNAs.

### CPEB4 binds to CPE variants upstream of polyadenylation sites

**Fig. 4:**
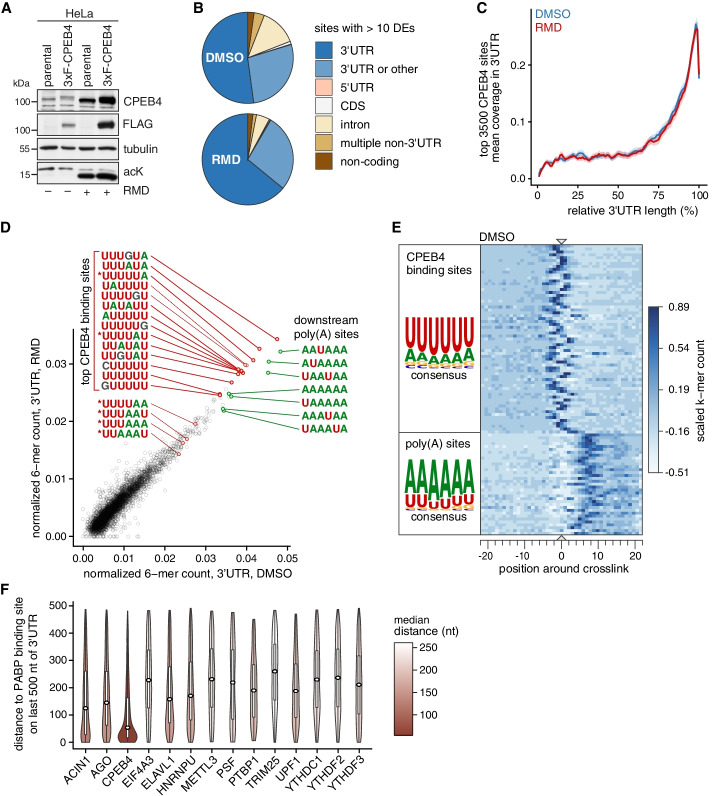
Identification of CPEB4 binding sites by PAR-CLIP. **A** Western blot analysis of CPEB4 protein expression in parental HeLa cells and HeLa cells expressing endogenously tagged 3xFLAG (3xF)-CPEB4. Cells were treated with 20 nM RMD or an equal volume of solvent (DMSO) for 16 h. **B** Distribution of transcript categories containing CPEB4 binding sites derived from PAR-CLIP experiments in DMSO- and RMD-treated HeLa-3xF-CPEB4 cells. Only sites with ≥ 10 diagnostic events (DEs), comprising the sum of T deletions and T-to-C conversions, are shown. Category “3′UTR or other” indicates overlap of a 3′UTR annotation and another category on the same strand. Category “multiple non-3′UTR” includes any number of overlapping annotation categories except 3′UTR. **C** Coverage profile of CPEB4 binding along the length of binned 3′UTRs. Plotted are equal length windows (50 nt) around crosslink centers of the 3500 top scoring CPEB4 binding sites over target 3′UTRs common for both DMSO- and RMD-treated conditions. Each 3′UTR is scaled to 100 bins. **D** Scatterplot showing normalized counts of the top 100 3′UTR-located 6-mers within 50-nt windows aligned at the crosslink centers in the DMSO- and RMD-treated condition. Counts were normalized by the 6-mer count in all 3′UTRs, and the sequence is shown for the most abundant 6-mers and 6-mers matching the canonical CPE (marked with *). **E** Heatmap showing scaled count of the 100 most abundant 6-mers in 50-nt windows around crosslink centers in DMSO-treated HeLa-3xF-CPEB4 cells. Each line represents one 6-mer. Color intensity corresponds to scaled frequency of this 6-mer over all 3′UTR CPEB4 binding sites. The position refers to the center (nt 3) of the 6-mers. On the left side, 6-mers were segregated by position into 2 clusters using k-means clustering, and the positional weight matrix is shown for each cluster. **F** Distribution of distances between 3′UTR binding sites of PABP and other RBPs including CPEB4. The distance from the center of the RBP binding site to the center of the closest downstream PABP binding site on the same 3′UTR, within 500 nt of the annotated 3′end, was calculated. CLIP data from this study (CPEB4, DMSO-treated condition) and published datasets (Additional file 7) were used

Our next goal was to explore the mRNA targets of CPEB4 under normal and hyperacetylated conditions. So far, binding of CPEB4 to mRNA was examined by RNA-IP from different cellular systems [40, 41, 44] and through identification of bindings sites *in vitro* by SELEX [69]. To identify cellular CPEB4 RNA binding sites and the spectrum of bound mRNAs more precisely, we set out to perform PAR-CLIP [70] based on the highly specific purification of endogenous CPEB4 using HeLa-3xF-CPEB4 cells described above. Importantly, CPEB4 expression levels were very similar in parental HeLa and HeLa-3xF-CPEB4 cells (Fig. 4A), and induction of CPEB4 by RMD was maintained in the tagged clone (Fig. 4A, Additional file 2: Fig. S3D).

PAR-CLIP enables the transcriptome-wide identification of RNA binding sites of RBPs and relies on the incorporation of the photoactivatable ribonucleoside 4-thiouridine (4sU) into nascent RNA. 4sU increases crosslinking efficiency and gives rise to mutations at the position of UV-induced crosslinks during reverse transcription, which can be used to pinpoint RBP contact sites with nucleotide resolution [71]. A disadvantage of 4sU labeling is that it can induce adverse effects such as nucleolar stress [72]. To minimize side effects from 4sU incorporation, we assessed the impact on stress kinase signaling and found that labeling for 2 h with 200 μM 4sU was below the threshold for p38-MAPK and c-Jun N-terminal kinase (JNK) activation (Additional file 2: Fig. S5A). Hence, we decided to perform PAR-CLIP under these conditions to identify the CPEB4 mRNA occupancy profile.

3xF-CPEB4 HeLa cells, and as negative control, parental HeLa cells were labeled with 200 μM 4sU for 2 h followed by crosslinking at 365 nm. CPEB4:RNA complexes were specifically enriched by IP using α-FLAG antibody and visualized by ^32^P labeling of RNA 5′ends, giving rise to a distinct signal after denaturing PAGE with no background in the parental HeLa cell sample (Additional file 2: Fig. S5B). Two additional PAR-CLIP replicates were performed with infrared dyes on the ligation adapters (Additional file 2: Fig. S5C), and the replicates were in good agreement with each other (Additional file 2: Fig. S5D). To call CPEB4 binding sites, we used the peak caller omniCLIP [73]. Specifically, omniCLIP makes use of replicate information and considers underlying mRNA expression levels. As background samples for peak calling, we used total RNA-Seq data of DMSO- and RMD-treated HeLa cells.

The PAR-CLIP reads contain information about crosslinked nucleotides, in the form of T-to-C transitions and T deletions [74] (Additional file 2: Fig. S5E), which we call diagnostic events (DEs). We ranked CPEB4 binding sites (Additional file 6) by the sum of DEs per site and observed that the proportion of binding sites falling within 3′UTRs was high for sites with ≥10 DEs (Fig. 4B, Additional file 2: Fig. S5F). Within 3′UTRs, CPEB4 preferentially binds close to the 3′end (Fig. 4C). CPEB4 is known to associate with the consensus CPE motif UUUUAU via its two RRMs situated in the conserved C-terminal half [42, 43]. In our PAR-CLIP analysis, the most abundant 6-mers associated with CPEB4 in 3′UTRs comprised a number of U-rich variants of the CPE, including the G-containing UUUGUA motif as the top hit (Fig. 4D). Notably, canonical CPE motifs such as UUUAAU and UUUUAU were present among the CPEB4-bound sequences, yet mostly far below other CPE variants. In addition, several A-rich sequences were strongly associated with CPEB4, corresponding to the canonical poly(A) site (AAUAAA) and related sequence motifs (Fig. 4D). We did not observe strong differences between DMSO and RMD conditions in terms of binding site distribution over transcripts, 3′UTR position, or CPEB4 motif specificity (Fig. 4B–D). Essentially, differences in CPEB4 binding between the two conditions largely reflected underlying changes in the abundance of mRNA targets caused by RMD treatment (Additional file 2: Fig. S6A).

When we aligned the bound sequences on their top crosslink site (defined as the position with the highest sum of DEs over all replicates), the U-rich CPEB4 binding motifs were present at the crosslink site, whereas the A-rich motifs were enriched immediately (5–10 nts) downstream of the crosslink (Fig. 4E, Additional file 2: Fig. S6B). This could also be observed at the level of individual target genes (Additional file 2: Fig. S7). Of note, since the analysis was performed on genomic windows, the A-rich motifs do not derive from the poly(A) tail but are embedded within the 3′UTR and most likely represent variants of the poly(A) site. To further explore this possibility, we tested whether CPEB4 binds close to the poly(A) tail by assessing its proximity to the poly(A) binding protein (PAPB). Using a published PABP CLIP dataset from HeLa cells [75], we compared the distribution of distances between the closest upstream CPEB4 binding site and a downstream PABP binding site provided both are present on the same 3′UTR. Compared to other RBPs for which CLIP datasets from HeLa cells are available (Additional file 7), CPEB4 bound much closer to PABP than any of the other RBPs tested, with a median distance of 54 nts (Fig. 4F). Taken together, our PAR-CLIP analysis revealed that CPEB4 preferentially binds to variants of canonical CPEs with a positional preference immediately upstream of A-rich motifs, representing canonical and non-canonical poly(A) sites.

### CPEB4 promotes degradation of IEG mRNAs

**Fig. 5:**
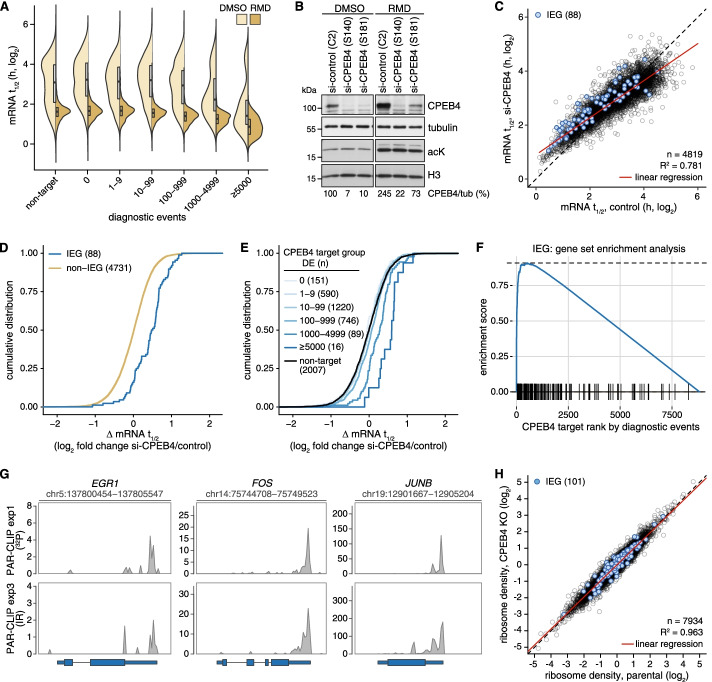
mRNA half-life analysis of CPEB4 target genes. **A** Transcriptome-wide mRNA decay was measured by RNA-Seq analysis of actinomycin D chase experiments in HeLa cells treated with 20 nM RMD or an equal volume of DMSO for 16 h. The graph depicts the distribution of mRNA half-lives grouped by the sum of diagnostic events in all CPEB4 binding sites per gene. **B** Western blot analysis of CPEB4 expression following KD with a control or two CPEB4-targeting siRNAs in HeLa cells. Cells were treated with 20 nM RMD or equal volume of DMSO for 16 h. **C** Scatter plot depicting mRNA half-lives determined by transcriptome-wide mRNA decay measurements in control vs. CPEB4 KD HeLa cells in the DMSO-treated condition. Average mRNA half-lives were calculated for the two CPEB4 siRNAs and for the two control conditions (control siRNA and untransfected HeLa cells). Immediate early gene (IEG) mRNAs are highlighted in blue. **D** The cumulative distribution of log_2_-transformed changes in mRNA half-lives upon CPEB4 KD is shown for a literature-curated set of IEGs and all other genes. **E** The cumulative distribution of log_2_-transformed changes in mRNA half-lives upon CPEB4 KD is shown for all genes grouped by the sum of diagnostic events in all CPEB4 binding sites per gene. **F** Gene set enrichment analysis showing enrichment of IEG mRNAs among CPEB4 target genes ranked by the sum of diagnostic events per gene. **G** Examples of CPEB4 PAR-CLIP coverage over the three IEGs *EGR1*, *FOS*, and *JUNB*. Only the longest gene model is depicted. **H** Scatter plot depicting the ribosome density of each mRNA based on Ribo-Seq analysis in CPEB4 KO clone #44 and parental HeLa cells (*n* = 2). IEG mRNAs are highlighted in blue

Given the mRNA destabilization observed by tethering of CPEB4 (Fig. 3D–G), our next goal was to assess the impact of CPEB4 on the stability of endogenous mRNAs. First, we compared CPEB4 target mRNAs identified by PAR-CLIP (Fig. 4, Additional file 8) with transcriptome-wide mRNA half-lives determined in HeLa cells by RNA-Seq at different time intervals after blocking transcription with actD. By assuming first-order decay kinetics and requesting a coefficient of determination (R^2^) > 0.5 for the analysis of log-transformed read counts by linear regression, reliable half-lives could be calculated for 4819 mRNAs (Additional file 9). When CPEB4 targets were ranked by the sum of DEs per gene, we observed a striking correlation whereby strong targets (>1000 DEs) have short mRNA half-lives under both DMSO and RMD conditions (Fig. 5A), indicating that the strength of CPEB4 binding is associated with rapid mRNA decay.

To determine if this association is dependent on CPEB4, we used the same approach to measure transcriptome-wide mRNA half-lives upon KD of CPEB4 with two different siRNAs (Additional file 9). Both siRNAs led to an efficient KD of CPEB4, though si-CPEB4 (S181) was slightly less potent than si-CPEB4 (S140, Fig. 5B). We combined the values from the two KD and two control samples, and while the overall stability of mRNAs was not affected (Additional file 2: Fig. S8A), the analysis revealed a distinct stabilization of short-lived mRNAs transcribed from immediate early genes (IEGs) in CPEB4 KD cells (Fig. 5C, D). Importantly, the degree of stabilization of specific mRNAs by CPEB4 KD correlated with the strength of CPEB4 binding as determined by the sum of DEs per gene (Fig. 5E). When looking at ranked target genes by gene set enrichment analysis, it was apparent that IEGs are strongly enriched in the top DE category (Fig. 5F). Examples of CPEB4 PAR-CLIP profiles are shown for three IEGs, *EGR1*, *FOS* and *JUNB* (Fig. 5G for whole gene overview, Additional file 2: Fig. S9 for detailed view at single base resolution).

Within our transcriptome-wide mRNA half-life dataset, IEG mRNAs were strongly stabilized upon KD of CPEB4 (Fig. 5C, D). A small stabilizing effect was also observed by KD of CPEB4 in RMD-treated cells (Additional file 2: Fig. S8B). This indicates that CPEB4 promotes IEG mRNA degradation also upon HDAC inhibition, though acetylation-induced turnover of global mRNA [57] appears to override most transcript-specific regulation.

AREs represent an abundant class of mRNA destabilizing elements in mammalian cells [76], and several IEG mRNAs were shown to undergo ARE-dependent degradation [77–81]. Since ARE-containing mRNAs were not stabilized upon CPEB4 KD (Additional file 2: Fig. S8C), CPEB4-mediated mRNA decay appears to be ARE-independent.

Given that CPEB4 is well known to promote cytoplasmic polyadenylation and translational activation [36, 40, 41, 44], we explored whether CPEB4 depletion would alter ribosome occupancy of IEG mRNAs. To this end, we performed ribosome profiling (Ribo-Seq) in control and CPEB4 knockout (KO) HeLa cells generated by CRISPR/Cas9-mediated genome editing. Characterization of selected CPEB4 KO clones #44 and #45 revealed that they fail to express CPEB4 due to an insertion (#44) or deletion (#45) downstream of the ATG start codon (Additional file 2: Fig. S10A-C). Ribo-Seq analysis was then carried out with the CPEB4 KO clone #44, using parental HeLa cells as control. Quality assessment showed pronounced periodicity of the footprint RNA (Additional file 2: Fig. S11A), as well as high reproducibility between biological replicates (Additional file 2: Fig. S11B). Overall, changes in ribosome density upon CPEB4 KO were very small and IEG mRNAs did not display systematic differences in their ribosome densities (Fig. 5H, Additional file 2: Fig. S11C; Additional file 10). Thus, in the examined cellular context, CPEB4 appears to regulate IEG mRNA stability rather than translation.

### IEG mRNAs are stabilized upon CPEB4 knockout

**Fig. 6:**
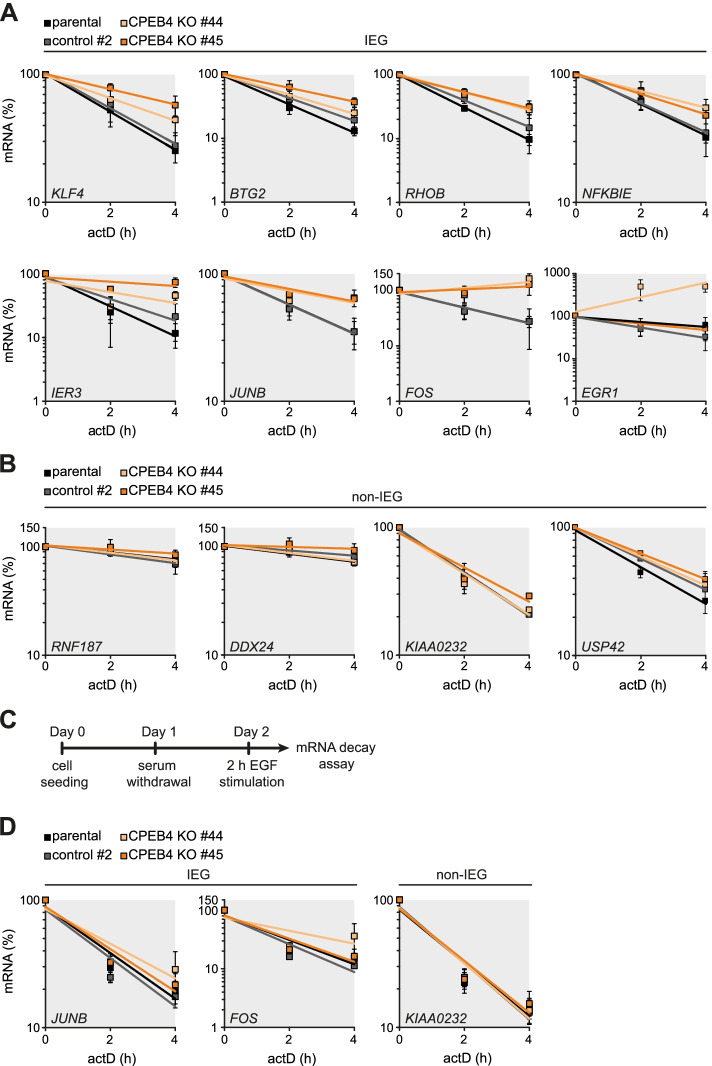
CPEB4-dependent IEG mRNA degradation. **A** Decay curves of eight IEG mRNAs containing CPEB4 binding sites were quantified in parental HeLa cells, control clone #2 and CPEB4 KO clones #44 and #45. Transcription was terminated by treatment of cells with 5 μg/ml actinomycin D, and total RNA was extracted after 0, 2, and 4 h. The mRNA levels were measured by qRT-PCR; 18S rRNA was used for normalization. Data are presented as mean ± SD (*n* = 3). **B** The same analysis as in **A** was conducted for four non-IEG mRNAs. **C** Schematic illustration of the experimental workflow used to measure IEG mRNA half-lives following mitogenic stimulation. HeLa cells were seeded at day 0 and serum-starved for 24 h before stimulation with 20 ng/ml human epidermal growth factor (EGF). Following 2 h of stimulation, mRNA stability was measured as described in **A**. **D** Decay curves of IEG mRNAs *JUNB*, *FOS*, and the non-IEG mRNA *KIAA0232* were quantified following stimulation of HeLa cell lines with EGF as described in **A** and **C**. Data are presented as mean ± SD (*n* = 5)

Using the HeLa CPEB4 KO cells, we then validated the role of CPEB4 in promoting the degradation of IEG mRNAs. Following transcriptional shut-off with actD, total RNA was extracted at regular time intervals from CPEB4 KO clones #44 and #45, parental HeLa cells as well as a CRISPR/Cas9 control clone. By qRT-PCR, we then determined the stability of eight IEG mRNAs (*KLF4*, *BTG2*, *RHOB*, *NFKBIE*, *IER3*, *JUNB*, *FOS*, *EGR1*), which we had identified as targets of CPEB4 (Additional file 11). As controls we used four non-IEG mRNAs that showed no (*RNF187*, *KIAA0232*) or weak (*DDX24*, *USP42*) CPEB4 binding in the PAR-CLIP analysis. With the exception of *EGR1* and *BTG2*, where mRNA stability was elevated only in one CPEB4 KO clone, all other IEG mRNAs were stabilized in both KO clones (Fig. 6A, Additional file 2: Fig. S12A). In contrast, none of the four non-IEG mRNAs showed a clear difference in mRNA stability between KO clones and the two controls (Fig. 6B, Additional file 2: Fig. S12B). Hence, KO of CPEB4 confirmed that IEG mRNAs are subject to CPEB4-mediated mRNA degradation.

IEGs are transcriptionally induced within minutes in response to growth factor stimulation, and many IEGs follow a transient expression pattern that is terminated by a combination of transcriptional shut-off and rapid mRNA decay [82]. To explore if CPEB4-mediated mRNA decay contributes to the phased expression pattern of IEGs, we chose epidermal growth factor (EGF), which is known to transiently induce IEG expression in HeLa cells [83]. The mRNA stability of two well-characterized IEGs, the proto-oncogenes *JUNB* and *FOS*, was examined in control and CPEB4 KO cells following EGF stimulation. Cells were subjected to serum deprivation for 24 h prior to stimulation with EGF for 2 h followed by subsequent transcriptional shut-off with actD (Fig. 6C). Both *JUNB* and *FOS* mRNA were induced 2-4-fold following exposure to EGF for 2 h, whereas the expression of non-IEG mRNAs *KIAA0232* and *GAPDH* remained unchanged (Additional file 2: Fig. S12C). In contrast to the unstimulated condition (Fig. 6A, Additional file 2: Fig. S12A), differences in *JUNB* and *FOS* mRNA stability were barely detectable between the two controls and CPEB4 KO clones upon EGF treatment (Fig. 6D, Additional file 2: Fig. S12D). Hence, CPEB4 promotes the degradation of IEG mRNAs under basal conditions, while it does not appear to have this function upon EGF stimulation.

### All CPEB family members interact with the CCR4-NOT complex but have different capacities to promote mRNA deadenylation and decay

**Fig. 7:**
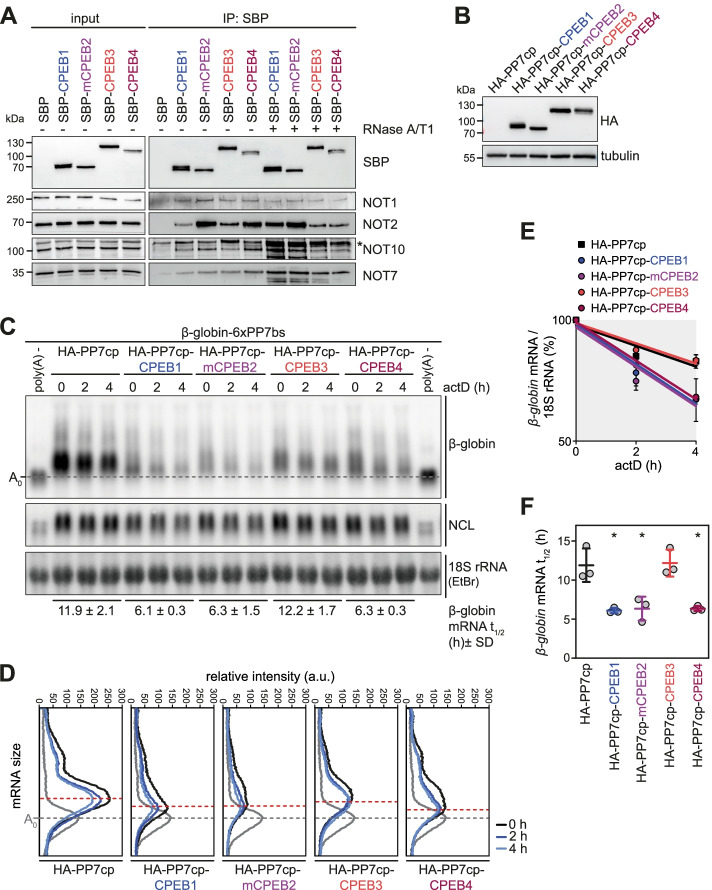
CPEB1-4 repressor complex formation by interaction with the CCR4-NOT complex. **A** Co-IP of endogenous NOT1, NOT2, NOT7, and NOT10 with ectopically expressed, SBP-tagged human CPEB1, CPEB3, CPEB4, and mouse (m) CPEB2, in HeLa cells. The IP was carried out using streptavidin beads in the absence and presence of 26 U RNase A/T1, and precipitated proteins were detected by western blot analysis using specific antibodies as indicated. The asterisk denotes a non-specific band. **B** Western blot analysis of HA-tagged human CPEB1, CPEB3, CPEB4, and mouse mCPEB2 fused to the bacteriophage PP7 coat protein (PP7cp) in HeLa cells. **C** For the tethering assay, HeLa cells were transiently transfected with HA-PP7cp, HA-PP7cp-CPEB1, -CPEB3, -CPEB4, or -mCPEB2 together with a *β-globin* reporter mRNA containing 6 repeats of the PP7bs. *β-globin* reporter mRNA decay and deadenylation were analyzed as in Fig. 3E. **D** Deadenylation was visualized by densitometric analysis of the *β-globin* mRNA signal. RNA digested with RNase H in presence of oligo-dT serves as a reference for fully deadenylated (poly(A)-) RNA. The signal intensity was plotted as a function of the poly(A) tail length and the maximum of the 4-h timepoint is indicated by a red dashed line. **E** Decay of *β-globin* mRNA in the tethering assay is depicted after normalization to 18S rRNA (mean values ± SD; *n* = 3). **F** Half-lives of the *β-globin* mRNA in the tethering assays were calculated following first-order decay kinetics (mean values ± SD; *n* = 3) and *p*-values were calculated using a two-sided, paired *t*-test; * *p* < 0.05. Note that the *β-globin* mRNA decay curve in **E** and the reporter mRNA half-life measured after transfection of HA-PP7cp-CPEB4 in **F** are the same as in Fig. 3F and G

We finally set out to perform a comparative analysis of all four CPEB family members with respect to their capacity in promoting mRNA decay via the CCR4-NOT complex. Co-IP experiments showed that SBP-tagged human CPEB1, CPEB3 and CPEB4 as well as mouse CPEB2 associate with endogenous NOT1, NOT2, NOT10 and NOT7/CAF1a (Fig. 7A). Importantly, none of these interactions was dependent on the presence of RNA (Fig. 7A, Additional file 2: Fig. S13A). These results indicate that all four CPEB proteins have the capacity to recruit the CCR4-NOT complex. Next, we compared the mRNA destabilizing activity of all four CPEBs using the tethering approach introduced in Fig. 3, expressing equal amounts of HA-tagged CPEBs together with the *β-globin* reporter mRNA (Fig. 7B). Human CPEB1, CPEB4 and mouse CPEB2 were equally potent in promoting deadenylation, accelerating *β-globin* reporter mRNA degradation by 2-fold (Fig. 7C–F). Notably, tethering of CPEB1 and mouse CPEB2 led to pronounced deadenylation, which was already evident at the 0-h timepoint (Additional file 2: Fig. S13B). In contrast, tethering of CPEB3 induced deadenylation only weakly and did not lead to detectable reporter mRNA decay (Fig. 7C–F, Additional file 2: Fig. S13B), indicating that an interaction between CPEBs and CCR4-NOT is not the sole determinant of their mRNA destabilizing activity. Our comparative analysis demonstrates that although all four CPEBs interact with the CCR4-NOT complex, there are differences in their ability to promote mRNA degradation as RNA adaptors within repressor complexes.

## Discussion

IEGs represent a unique class of genes characterized by rapid induction, which occurs typically within 30 minutes of a stimulatory input, does not require *de novo* protein synthesis, and is meditated by dedicated transcription factors including SRF, NF-κB and CREB [84]. A second characteristic feature of IEGs is their transient expression pattern, which involves transcriptional shut-off and rapid degradation of IEG mRNAs, often also combined with rapid degradation of the protein product [82]. The short-lived nature of IEG mRNAs depends on a variety of *cis*-acting sequence elements such as AREs [77, 85], stem-loop forming constitutive decay elements [30], coding region determinants of instability [77] as well as micro RNA binding sites [86, 87].

Here, we discovered that CPEB4 preferentially binds to IEG mRNAs (Fig. 5F, G), and that a large proportion of IEG mRNAs are subject to CPEB4-mediated mRNA degradation (Figs. 5C, D and 6A). In fact, out of the 102 IEG mRNAs for which we could determine half-lives in the DMSO condition, 29 are strongly bound by CPEB4 (as defined by ≥ 1000 DEs per gene), and 19 of those are stabilized ≥1.5-fold by KD of CPEB4 in the DMSO condition (Additional file 11). Thus, CPEB4 binding acts as a determinant of IEG mRNA instability.

So far, CPEB4 was primarily known as part of an activator complex that enhances translation via poly(A) tail lengthening of selected target mRNAs during meiotic and mitotic cell division [36–38], cancer progression [40, 41] and neurodevelopment [88]. Our results show that CPEB4 has an additional important function as part of a repressor complex that destabilizes specific mRNAs through its ability to associate with the CCR4-NOT deadenylase complex (Fig. 3A). Notably, mRNA instability increases with the strength of CPEB4 binding (Fig. 5A), which hence serves as a critical determinant for functional target prediction of the repressor complex. In line with a parallel study by Duran Arqué et al*.* [89], we confirmed that all four CPEB proteins can form a CCR4-NOT-associated repressor complex (Fig. 7A). Complementary to the observation that CPEB1 and CPEB2-4 form two subfamilies whose cytosolic aggregation in liquid-like droplets is differentially regulated by phosphorylation [89], we demonstrate that CPEBs display functional differences in their ability to promote mRNA deadenylation and decay (Fig. 7C–F, Additional file 2: Fig. S13B). Among all four CPEBs, CPEB3 was found to have the lowest tendency of cytoplasmic foci formation [89] and displayed the weakest mRNA destabilizing activity in our tethering approach (Fig. 7C–F). These findings support a direct connection between CPEB phase separation behavior and their activity within CCR4-NOT repressor complexes. Our observations are also in line with another recent study showing that CPEB4 acts as a repressor of transcription factor expression in the heart, acting both at the level of translation and mRNA decay [90].

Akin to CPEB1 and CPEB3 [45, 46], we found that the interaction between CPEB4 and the CCR4-NOT complex depends on the general mRNA decay factor TOB1 (Fig. 3B), which interacts with poly(A)-bound PABP via its two C-terminal PAM2 motifs [91] and recruits the CCR4-NOT complex via its N-terminal APRO domain [92]. Thus, our results support a model where CPEB4 mRNA binding provides specificity for TOB1-mediated recruitment of the CCR4-NOT complex. Since CPEB4 can still co-precipitate small amounts of NOT1 and NOT7/CAF1a in the absence of TOB1 (Fig. 3B), other members of the BTG/TOB family might fulfill redundant functions in bridging CPEB4 with the CCR4-NOT complex. CPEB3 and CPEB4 were shown to directly bind TOB1, whereby the interaction of CPEB3 was found to depend on its C-terminal RNA-binding domain (RBD) [45] comprising two RRMs and two zinc fingers. Since the RBDs of CPEB3 and CPEB4 share >95% amino acid sequence identity, their interaction with TOB1 is most likely conserved.

Our PAR-CLIP analysis revealed that CPEB4 mRNA binding sites comprise a range of U-rich sequence motifs (Fig. 4D). Notably, canonical CPEs [93] are among the CPEB4 binding motifs we identified, but do not represent the most abundant motifs. Rather, we found strongest binding to the G-containing CPE variant UUUGUA. These results are consistent with CPEB4-binding motifs previously identified by in vitro selection approaches [69, 94]. It is important to note that our PAR-CLIP analysis relies on binding of CPEB4 expressed from the endogenous gene locus to target mRNAs inside cells. Since all binding partners are expressed at their physiological levels in this system, our results are likely to represent a highly accurate picture of the CPEB4 binding landscape.

A second outcome from our PAR-CLIP analysis is that CPEB4 preferentially binds near the end of 3′UTRs (Fig. 4C), in close proximity to PABP binding sites (Fig. 4F) and upstream of internal A-rich motifs containing 0, 1, or 2 Us, including the canonical poly(A) signal AAUAAA (Fig. 4D, E; Additional file 2: Fig. S6B). Based on their position, we concluded that these A-rich motifs represent canonical and non-canonical poly(A) sites. Notably, the G-containing CPE variant UUUGUA followed by an A-rich poly(A) site motif corresponds exactly to the consensus motif UUUUGUAUAAAA identified for CPEB2-4 through RNA immunoprecipitation in a parallel study published by Duran Arqué et al. [89]. These findings are consistent with the previously documented interaction of CPEB proteins with the cleavage and polyadenylation specificity factor (CPSF) complex, which recognizes poly(A) sites [38, 95, 96]. This mechanism is ideally suited to place CPEB4 close to the end of 3′UTRs and begs the question whether binding of CPEB4 to target mRNAs occurs in the nucleus.

While the effect of CPEB4 KD on IEG mRNA stability was much stronger under control (DMSO) conditions, a similar effect—though weaker—was also evident when cells were treated with RMD (Additional file 2: Fig. S8B). This result indicates that CPEB4 also acts as an mRNA destabilizing adaptor protein following an increase in cellular acetylation. Hence, induction of CPEB4 expression by HDAC inhibitors (Fig. 2) contributes to acetylation-induced degradation of poly(A) mRNA [57], though only for a subgroup of mRNAs. Additional RBPs such as STAU1 and HELZ may participate in the accelerated degradation of other mRNA subgroups since these two proteins show enhanced binding to poly(A) RNA upon RMD treatment (Fig. 1B, D) and are known to promote mRNA degradation via different pathways [97, 98].

In meiotic and mitotic cells, CPEB4 was found to be multiply phosphorylated within its N-terminal low complexity region through CDK1- and ERK2-mediated phosphorylation, which enhances its ability to promote cytoplasmic polyadenylation, and reduces its propensity to oligomerize and phase-separate into liquid-like droplets [99]. More specifically, *X. laevis* CPEB4 was shown to undergo ERK2-mediated phosphorylation at 7 serine residues, 6 of which are fully conserved in human CPEB4 (Additional file 2: Fig. S14) [99]. Since ERK2 is activated following 2 h of EGF stimulation (Additional file 2: Fig. S12E), we propose that an increase in CPEB4 phosphorylation might shift the equilibrium towards the formation of an activator complex, thus explaining that CPEB4 no longer acts as a destabilizer of IEG mRNAs in EGF-treated cells (Fig. 6C).

Apart from the regulation of CPEB activity through phosphorylation, the distance between the CPE and the poly(A) site was shown to be part of a combinatorial code that determines whether specific mRNAs are translationally activated (polyadenylated) or repressed (deadenylated) during meiotic progression of *Xenopus* oocytes [100]. Moreover, CPEB4 was recently found to stabilize CPE- and ARE-containing cytokine mRNAs in macrophages [101], indicating that cell-type-dependent differences may affect the function of CPEB4 within post-transcriptional regulatory complexes controlling the stability of selected mRNA subsets. In the future, it will be interesting to determine more precisely the molecular events that allow switching between the polyadenylation-promoting CPEB4-activator complex and the CPEB4-repressor complex associated with CCR4-NOT-mediated mRNA deadenylation.

## Conclusions

In contrast to its widely accepted role as an activator of mRNA translation of selected target genes, we here describe an additional function of CPEB4 as part of a repressor complex that destabilizes specific mRNAs. Albeit displaying differences in their capacity to promote mRNA decay, recruitment of the CCR4-NOT complex is shared by all four members of the CPEB family. Using PAR-CLIP and transcriptome-wide mRNA half-life measurements, we demonstrate that CPEB4 binds to IEG mRNAs and promotes their degradation. Our study expands the repertoire of RBPs regulating IEG mRNA expression and shows that CPEB4 contributes to accelerated mRNA turnover upon HDAC inhibition via recruitment of the evolutionarily conserved CCR4-NOT deadenylase, thereby forming a repressor complex that accelerates the degradation of highly important mRNAs.

## Methods

### Cell culture and transfection

HeLa cells (a kind gift from Paul Anderson, Harvard Medical School) were cultured in Dulbecco’s modified Eagle’s medium (DMEM) supplemented with 10% (v/v) FBS (Sigma), 2 mM L-glutamine, 100 U/ml penicillin, and 0.1 mg/ml streptomycin (all PAN Biotech) and incubated at 37 °C in a 5% CO_2_ incubator. HeLa cells were authenticated via SNP profiling by Multiplexion GmbH at DKFZ and regularly tested for mycoplasma contamination using a PCR Mycoplasma Test Kit (AppliChem).

### Reverse siRNA transfection

Knock-down experiments were performed with Lipofectamine RNAiMAX transfection reagent (Thermo Fisher Scientific) and reverse transfection. siRNAs were used at a final concentration of 100 nM and diluted in Opti-MEM reduced serum medium (Thermo Fisher Scientific). Cells were transfected with siRNA over a period of 48 h. siRNAs were designed with help of the siDESIGN Center (Horizon Discovery), synthesized by Eurofins Genomics, and correspond to the following sequences (sense strand):

- S014 (C2, negative control),5′-GGUCCGGCUCCCCCAAAUGdTdT-3′;
- S075 (AllStars negative control siRNA, Qiagen), sequence unknown;
- S171 (TOB1),5′-CGUGGAUGAUAAUAAUGAAdTdT-3′;
- S140 (CPEB4-1),5′-CTGCCTCATTTGGCGAATAdTdT-3′;
- S181 (CPEB4-2),5′-GGGATTACGGGTTTGGAGTdTdT-3′;
- S068 (HDAC1)5′-AAGCCUCACCGAAUCCGCAdTdT-3′

### CRISPR/Cas9-mediated genome editing

All guide RNA sequences were designed in silico by using CRISPR design tools (https://zlab.bio/guide-design-resources). Guide RNA sequences correspond to the following sequences:

- CPEB4 (guide A),5′-TAAATGGGGGATTACGGGTT-3′ (top strand);
- CPEB4 (guide B),5′-GATAATAAATGGGGGATTAC-3′ (top strand)

For endogenous tagging of *CPEB4*, HeLa cells were transfected with an equal amount of Cas9-guide RNA expressing constructs pSpCas9(BB)-2A-GFP-*CPEB4*-guideA (p3646) and pSpCas9(BB)-2A-GFP-*CPEB4*-guideB (p3647) together with 100 pmol of a single-stranded donor oligonucleotide (5′-AAG GCG TGA GAC ATC AGG TTG TCA TTT TTT ATT GTG AGA TTC TGC TCC TAA AGA TAA TAA ATG GAC TAC AAG GAC CAC GAC GGC GAT TAT AAG GAT CAC GAC ATC GAC TAC AAA GAC GAC GAT GAC AAG GGA TCC GGC GAC TAC GGC TTT GGA GTG CTA GTG CAA AGC AAT ACT GGG AAT AAA TCT GCT TTT CCA-3′; IDT) using Lipofectamine 3000. Cells were treated with 10 μM RS-1 (Sigma-Aldrich) for 24 h to enhance CRISPR/Cas9-mediated genome editing efficiency. Two days after transfection, GFP^+^ cells were enriched by FACS (FlowCore, Medical Faculty Mannheim). Single clones were obtained by limiting dilution cloning using 96-well plates and 10% (v/v) conditioned medium. Integration of the 3xFLAG tag in single clones was monitored by western blotting and sequencing of the genomic locus.

To generate single CPEB4 KO clones, HeLa cells were transfected with an equal amount of Cas9-guide RNA expressing constructs p3646, p3647 and were further processed as described above. Successful CRISPR/Cas9-mediated genome editing in single clones was monitored by western blotting and sequencing of the genomic locus.

### Poly(A) RNA interactome capture

The interactome capture protocol was adapted from [59, 60]. Cells were chemically crosslinked as described previously [30]. Briefly, 1.6×10^6^ HeLa cells were seeded into 15-cm dishes. Following treatment with 20 nM RMD or solvent (DMSO) for 16 h, cells were washed twice with 1× PBS and crosslinked with 0.05% (v/v) formaldehyde in 1× PBS for 10 min at room temperature (RT) on a shaking platform. Excess formaldehyde was quenched by addition of 0.25 M glycine pH 7.0 for 5 min at RT. Cells were harvested by scraping in ice-cold 1× PBS and harvested by centrifugation at 400*g* for 3 min at 4 °C. Afterwards, cells were washed once with ice-cold 1× PBS and immediately flash-frozen in liquid nitrogen. Cells were mechanically disrupted using a Tissuelyzer II (4× 15 s, 25 Hz) in conjunction with 5 mm stainless steel beads (both from Qiagen). Cell powder was solubilized in IC lysis buffer (20 mM Tris-HCl pH 7.5, 500 mM LiCl, 1 mM EDTA, 5 mM DTT) supplemented with cOmplete EDTA-free Protease Inhibitor Cocktail (Roche). Lysates were vortexed briefly and incubated on a rotating shaker for 10 min at 4 °C. Lysates were cleared by centrifugation at 425*g* for 5 min at 4 °C. One percent of the total lysate was saved as input. An equal amount of total protein was used for poly(A) RNA affinity purification with oligo d(T)_25_ magnetic beads (NEB) for 1 h at 4 °C while rotating. Beads were washed 1× 5 min with lysis buffer, 2× 5 min with IC wash buffer 1 (20 mM Tris-HCl pH 7.5, 500 mM LiCl, 0.5% [w/v] LiDS, 1 mM EDTA, 5 mM DTT), 4× 5 min with IC wash buffer 2 (20 mM Tris-HCl pH 7.5, 500 mM LiCl, 0.1% LiDS [w/v], 1 mM EDTA, 5 mM DTT), and 2× 5 min with IC wash buffer 3 (20 mM Tris-HCl pH 7.5, 200 mM LiCl, 0.1% [w/v] LiDS, 1 mM EDTA, 5 mM DTT). After each washing step, beads were separated in a magnetic stand, resuspended in wash buffer, and incubated for 5 min at 4 °C on a rotating shaker. Ten percent of the beads were subjected to RNA purification, while 90% were used for protein analysis. Before RNA recovery using PeqGold TriFast (Peqlab), pull-down samples were digested with 10 μg/ml proteinase K (Promega) for 1 h at 37 °C in proteinase K digestion buffer (25 mM Tris-HCl pH 7.5, 2.5 mM CaCl_2_, 1.25 mM MgCl_2_). Prior to protein elution with 2× SDS sample buffer (100 mM HEPES pH 7.4, 4% [w/v] SDS, 20% [v/v] glycerol, 200 mM DTT, bromophenol blue), pull-down samples were digested with 13 U RNase A (Sigma-Aldrich) and RNase T1 (Thermo Fisher Scientific) in 1× RNase buffer (10 mM Tris-HCl pH 7.5, 150 mM NaCl, 0.05% [v/v] NP-40, 0.5 mM DTT) for 1 h at 37 °C. Protein samples were analyzed by the Mass Spectrometry and Proteomics Core Facility of the ZMBH (Center for Molecular Biology, Heidelberg University) or by western blotting as described below.

### Mass Spectrometry (LC-MS/MS)

Proteins from poly(A) RNA IC were shortly run into a 10% NuPAGE polyacrylamide Bis-Tris gel using 1× NuPAGE MOPS SDS Running Buffer (both from Thermo Fisher Scientific). The gel was stained with colloidal Coomassie (VWR) and subjected to MS analysis at the Mass Spectrometry and Proteomics Core Facility of the ZMBH (Center for Molecular Biology, Heidelberg University). Following in-gel reduction, alkylation, and trypsin digestion, LC-MS2 analysis was performed using a nanoHPLC liquid chromatography system directly coupled to an Orbitrap Elite mass spectrometer (both Thermo Fisher Scientific). Raw files were processed using MaxQuant version 1.5.3.30 [102] for peptide identification and quantification. MS2 spectra were searched against the Uniprot human_201605_UP000005640_9606 proteome database and the contaminants database by the Andromeda search engine. Carbamidomethylation of cysteine residues was defined as fixed modification, and N-terminal acetylation, oxidation of methionine, and deamidation of aspartic and glutamic acid were defined as variable modifications. Trypsin was selected as the proteolytic enzyme. Up to two missed cleavages were allowed. The maximum false discovery rate for proteins and peptides was 0.01, and a minimum peptide length of 7 amino acids was required.

### Analysis of mass spectrometry data

Downstream data analysis was performed using LFQ values in R v3.6.3. Commonly occurring contaminants and proteins matching the reversed part of the decoy database were excluded. To remove redundant protein entries, only LFQ values assigned to proteins or protein groups with the highest number of peptide counts (razor/unique) were considered. Protein entries without assigned LFQ values in both DMSO and RMD conditions were excluded from the analysis. To retain protein entries lacking mean LFQ values in only one condition (DMSO or RMD), intensity measurements of 0 were replaced with a positive real number (indicated as “not detected” in Fig. 1B). For the identification of proteins with differential binding to poly(A) RNA upon RMD treatment, only proteins enriched >10-fold over non-crosslinked controls were considered. Candidate proteins were identified based on an at least 2-fold increase or decrease in their LFQ intensity upon RMD treatment across two biological replicate experiments.

### RNA recovery and precipitation

Samples from poly(A) RNA interactome capture were mixed with 500 μl PeqGold TriFast (Peqlab). Following incubation at RT for 5 min, 100 μl chloroform was added. Samples were inverted 15 s by hand and subsequently centrifuged at 12,000*g* for 10 min at 4 °C. The upper aqueous phase was transferred to a fresh tube and mixed with 500 μl isopropanol and 1 μl GlycoBlue coprecipitant (Thermo Fisher Scientific). RNA was precipitated O/N at −20 °C. RNA was pelleted at 12,000*g* for 20 min at 4 °C. Precipitated RNA was washed with 1 ml ice-cold 75% (v/v) EtOH and centrifuged for 5 min at 12,000*g* and 4 °C. RNA was air-dried, resuspended in 10 μl RNase-free water, and dissolved at 65 °C for 10 min.

### PAR-CLIP

The PAR-CLIP protocol was adapted from [103, 104]. 3×10^6^ HeLa cells were plated in 15 cm dishes. On the following day, 20 nM RMD or an equal volume of solvent (DMSO) was added for 14 h followed by subsequent labeling with 200 μM 4sU (Sigma-Aldrich) for 2 h. Cells were crosslinked at 365 nm and 150 mJ/cm^2^ on ice, scraped in ice-cold 1× PBS, washed once in cold 1× PBS, and flash-frozen in liquid nitrogen. Depending on the condition, about 20–60 plates were used per PAR-CLIP experiment. Cell pellets were lysed on ice in iCLIP lysis buffer (50 mM Tris-HCl pH 7.4, 100 mM NaCl, 1% [v/v] Igepal CA-630, 0.1% [w/v] SDS, 0.5% [w/v] sodium deoxycholate, 40 U/ml RNasin) supplemented with cOmplete EDTA-free Protease Inhibitor Cocktail (Roche). Cell lysates were cleared by centrifugation. 0.1 U/μl RNAse I (Thermo Fisher Scientific) was used to digest the cell lysate in a 37 °C water bath for 3 min with manual mixing. For each IP, 20 μg of FLAG M2 antibody (Sigma-Aldrich) was coupled to 100 μl Dynabeads Protein G (Thermo Fisher Scientific). Protein-RNA complexes were immunoprecipitated for 2 h at 4 °C. Beads were washed twice with iCLIP high salt buffer (50 mM Tris-HCl pH 7.4, 1 M NaCl, 1 mM EDTA, 1% [v/v] Igepal CA-630, 0.1% [w/v] SDS, 0.5% [w/v] sodium deoxycholate) and three times with iCLIP PNK buffer (20 mM Tris-HCl pH 7.4, 10 mM MgCl_2_, 0.2% [v/v] Tween-20). Following dephosphorylation with T4 PNK (NEB) in PNK buffer (70 mM Tris-HCl, pH 6.5, 10 mM MgCl_2_, 1 mM DTT) for 15 min, beads were further processed as follows depending on whether the radioactive (^32^P-CLIP) or infrared (irCLIP) protocol was used. For ^32^P-CLIP, beads were labeled with 0.5 μCi/μl [γ-^32^P]-ATP for 30 min at 37 °C. Following two washes with iCLIP PNK buffer, beads were resuspended in NuPAGE LDS sample buffer (Thermo Fisher Scientific) and incubated for 10 min at 70 °C. RNA-protein complexes were resolved on NuPAGE 3-8% Tris-acetate gels (Thermo Fisher Scientific) followed by transfer to nitrocellulose membranes (VWR) using 2× NuPAGE transfer buffer (Thermo Fisher Scientific). RNA-protein complexes were visualized using a Phosphorimager screen (GE Healthcare), cut out, and isolated from the membrane by proteinase K digestion in iCLIP PK buffer (100 mM Tris-HCl pH 7.4, 50 mM NaCl, 10 mM EDTA, 7 M urea). RNA was extracted via phase separation with phenol:chloroform:isamylalcohol (25:24:1; AppliChem). RNA was size-selected by 15% TBE-urea PAGE and used for library preparation with the NEXTflex Small RNA-Seq Kit v3 (Perkin Elmer). For irCLIP, ligation of a 3′adapter conjugated to an 800-nm infrared dye (/5rApp/NNN NTG GAA TTC TCG GGT GCC AAG GAA AAA AAA AAA A/iAzideN/AAA AAA, IDT) was carried out in a reaction containing 1 pmol IR 3′adapter, 10% (w/v) PEG8000, 200 U Rnl2K227Q (NEB), 10 U SuperaseIn (Thermo Fisher Scientific), and 5 mM DTT in 1× T4 RNA ligase buffer (NEB) at 16 °C overnight. Following two washes with iCLIP PNK buffer, phosphorylation was carried out for 20 min at 37 °C in a reaction containing 20 U T4 PNK (NEB), 10 U SuperaseIn, 1 mM ATP, 5 mM DTT. Ligation of a 5′adapter conjugated to a 680-nm infrared dye (/5AzideN/GTT CAG AGT TCT ACA GTC CGA CGA TCC TGA TCrNrNrNrNrNrNrN) was carried out in a reaction containing 1 pmol 5′adapter, 25% (w/v) PEG8000, 30 U Rnl1 (NEB), 1 mM ATP, 10 U SuperaseIn, and 5 mM DTT in Rnl1 buffer (NEB) at 25 °C for 2 h. Following two washes with iCLIP PNK buffer, beads were resuspended in NuPAGE LDS sample buffer (Thermo Fisher Scientific) and incubated for 10 min at 70 °C. RNA-protein complexes were resolved on NuPAGE 4-12% Bis-Tris gels (Thermo Fisher Scientific) along with a near-infrared protein ladder, followed by transfer to nitrocellulose membranes. RNA-protein complexes were visualized on a Typhoon imaging device (GE Healthcare), cut out of the membrane, and extracted by digestion with 10 U proteinase K (Thermo Fisher Scientific) in SDS-proteinase K buffer (10 mM TrisCl pH 7.5, 5 mM NaCl, 0.1 mM EDTA, 0.02% [w/v] SDS) at 55 °C for 45 min. Ligated RNA was purified via oligo-dT magnetic beads (Thermo Fisher Scientific) and reverse transcribed as previously described [104]. NEBNext small RNA primers were used to prepare the sequencing library from cDNA. Both types of CLIP libraries were multiplexed and sequenced on a NextSeq550 device (Illumina). Sequencing of infrared CLIP libraries was performed together with a PhiX control library.

### PAR-CLIP data analysis

Following demultiplexing, PAR-CLIP reads were processed in the following way. Adapter sequences were removed with Cutadapt [105]. UMI-tools [106] was used to extract unique molecular identifiers (UMIs) from the reads and to deduplicate reads after alignment. Sequences mapping to PhiX were removed with bowtie [107]. The remaining reads were aligned to the hg19 version of the human genome (Gencode v19) with a combination of STAR [108] and segemehl [109] aligners. More precisely, the reads with diagnostic deletion events were soft-clipped by the STAR aligner. Those reads were extracted and re-mapped with segemehl, which is able to align short reads with deletions. Aligned reads were further filtered to include only uniquely mapping reads. Those reads were used for peak calling with omniCLIP [73]. As background for omniCLIP peak calling, all three replicates of the DGE dataset were used. The raw peak calls from omniCLIP were imported into R (https://www.R-project.org/) and analyzed using the following R and Bioconductor packages:

- tidyverse [110]
- reshape2 [111]
- Hmisc (https://CRAN.R-project.org/package=Hmisc)
- magrittr (https://CRAN.R-project.org/package=magrittr)
- ggplot2 [112]
- lattice [113]
- ggrepel (https://CRAN.R-project.org/package=ggrepel)
- ggpubr (https://CRAN.R-project.org/package=ggpubr)
- ggunchained (https://github.com/supplyandcommand/ggunchained)
- cowplot (https://CRAN.R-project.org/package=cowplot)
- gridGraphics (https://CRAN.R-project.org/package=gridGraphics)
- magick (https://CRAN.R-project.org/package=magick)
- rio (https://cran.r-project.org/package=rio)
- extrafont (https://CRAN.R-project.org/package=extrafont)
- DESeq2 [114]
- BSgenome.Hsapiens.UCSC.hg19 (https://bioconductor.org/packages/BSgenome.Hsapiens.UCSC.hg19/)
- plyranges [115]
- Biostrings (http://www.bioconductor.org/packages/Biostrings/)
- GenomicRanges and GenomicFeatures [116]
- rtracklayer [117]
- Rsamtools (http://bioconductor.org/packages/Rsamtools)
- motifStack [118]
- Gviz [119]
- ellipse (https://CRAN.R-project.org/package=ellipse)
- genomation [120]
- RCAS [121]
- SRAdb [122]
- fgsea [123]

First, a non-overlapping (reduced) set of peaks was generated. To count diagnostic events (i.e., sum of T-to-C conversions and T deletions), Rsamtools pileup was used. T-to-C transitions and T deletions were summed up per peak and per gene to rank peaks and genes by the total number of diagnostic events. The coordinate within each peak with the highest sum of all diagnostic events (T deletions and T-to-C conversions) across three replicates was taken as the crosslink center for that peak. The GenomicRanges package was used to intersect peaks with transcript regions as per Gencode v19 annotation. The RCAS package functions were used to calculate and plot coverage profiles over 3′UTRs. To visualize the top 50 most abundant 6-mers in 50-nt crosslink-centered regions, 6-mers were counted using the oligonucleotideFrequency function within the Biostrings package. Heatmaps were made with functions from the genomation package. Differential expression analysis was performed with the DESeq2 package. Gene set enrichment analysis (GSEA) was performed using the fgsea package. The source of ARE transcripts was the database according to [76] and for IEG transcripts the literature compilation according to Arner et al. [124]. To analyze distances to other RBPs, the raw CLIP data from HeLa cells (listed in Additional file 7) were uniformly processed with the universal mapping pipeline (https://github.com/slebedeva/CLIP_mapping). omniCLIP peaks were called for all HeLa RBPs against the same DGE background as was used for CPEB4. The distance was calculated between the center of the RBP peak and the center of the PABP peak if both were located in the last 500 nts of the same 3′UTR. Details of the scripts can be found at https://github.com/slebedeva/CPEB4_public [125].

### Co-IP

Cells were harvested by scraping in ice-cold 1× PBS, centrifuged at 400*g* for 3 min at 4 °C, and immediately flash-frozen in liquid nitrogen. Cryogenic lysis with a Tissuelyzer II was performed as described above. Cell powder was solubilized in lysis buffer (20 mM Tris-HCl pH 7.5, 150 mM NaCl, 1.5 mM MgCl_2_, 1 mM DTT, 0.05% [v/v] NP-40) supplemented with cOmplete EDTA-free Protease Inhibitor Cocktail (Roche). Lysates were vortexed briefly and incubated on a rotating shaker for 10 min at 4 °C. Lysates were cleared by centrifugation at 425*g* for 5 min at 4 °C. Protein concentration was determined by Bradford assay (Bio-Rad). One percent of the total lysate was saved as input. Equal amounts of total protein were used for IP using Dynabeads MyOne Streptavidin C1 (Thermo Fisher Scientific) for 3 h at 4 °C on a rotating shaker. Beads were washed once with ice-cold lysis buffer and six times with ice-cold wash buffer (20 mM Tris-HCl pH 7.5, 150 mM NaCl, 2.5 mM MgCl_2_, 1 mM DTT, 0.1% [v/v] NP-40). Bound proteins were eluted with 2× SDS sample buffer.

### mRNA decay assay

HeLa cells were washed once with pre-warmed 1× PBS and trypsinized. Cells were resuspended in regular growth medium containing 5 μg/ml actinomycin D (AppliChem), and harvested at four consecutive time points (0, 2, 4, 6 h). Culture medium was aspirated, and cells were immediately flash-frozen in liquid nitrogen. RNA isolation was performed using the Universal RNA Purification Kit (EURx/Roboklon) including an on-column DNase digestion step. The RNA was analyzed by northern blotting, qRT-PCR, or RNA-Seq as described below. mRNA half-lives were calculated assuming first-order decay kinetics. mRNA abundance was normalized to 18S rRNA and plotted against time. Curves with the following equation were fitted to the data points by linear regression: *y*=*a* ×*e*^(*b* × *t*)^, where *y* stands for the relative mRNA signal and *t* for the time. mRNA half-lives were calculated as follows: *t*_1/2_= ln(2) / −b.

### RNase H digestion

To remove poly(A) tails from the 3′end of mRNAs, 10 μg total RNA was mixed with 3 μg oligodT_18_. RNA:DNA duplexes were generated in a thermocycler according to the following program: 3 min at 70 °C, 5 min at 42 °C, ramp down to 25 °C with 0.1 °C/s. RNase H digestion was performed using 5 U RNase H (NEB) for 20 min at 37 °C in the presence of 40 U RNasin (Promega). RNA was recovered by phase separation using phenol:chloroform:isamylalcohol (25:24:1; AppliChem) and precipitated following addition of 1 μl GlycoBlue coprecipitant (Thermo Fisher Scientific), 0.1 volumes 5M NaOAc, and 2.5 volumes 100% EtOH. RNA was precipitated O/N at −20 °C and further processed as described above.

### Transcriptome-wide mRNA half-life measurements

Prior to rRNA depletion using the Ribo-Zero Gold Kit (Illumina), 1 μg total RNA was mixed with 2 μl 1:100 diluted ERCC RNA Spike-In Mix (Thermo Fisher Scientific). Libraries were prepared with the NEBNext Ultra II Directional RNA Kit (NEB). Samples were equimolarly pooled and sequenced on a NextSeq550 system (Illumina) with 75 bases single end. Reads were mapped to the human genome (Gencode V27 as downloaded from UCSC Genome browser wgEncodeGencodeBasic27 table) and the ERCC92 spike-in mix with STAR v2.5.3a [126], allowing up to 2 mismatches and chimeric read detection with a minimum of 10 nt per segment (--chimSegmentMin 10). Read counts were summarized at the gene level with the featureCounts function of the subread package v1.6.3 [127]. A read was only counted when it was completely contained within an exon. Calculation of mRNA half-lives was performed using an in-house-developed R script as previously described [58]. Briefly, read counts were divided by the sum of all reads assigned to the spike-in sequences. Linear regression was performed on log-transformed read counts. Half-lives were calculated from the slope of the regression line with the equation: ln(2) / -slope. Only genes with a positive half-life and a coefficient of determination *R*^2^ > 0.5 in all conditions were used for subsequent analyses.

### Differential gene expression analysis

Using the Ribo-Zero Gold Kit (Illumina), 650–750 ng total RNA was subjected to rRNA depletion. Libraries were prepared with the TruSeq Stranded Total RNA Library Prep Kit (Illumina). Samples were equimolarly pooled and sequenced on a HiSeq 2500 device (Illumina) with 50 bases single end by the CellNetworks Deep Sequencing Core Facility (Heidelberg University). Reads were mapped with Bowtie v1.0.0 [107] to the human transcriptome (Gencode V19 transcriptome as downloaded from the UCSC Genome Browser wgEncodeGencodeBasicV19 table), allowing a maximum of two mismatches and reporting all alignments in the best stratum. Read counts were summarized at the gene level with an in-house-developed R script discarding all reads that map to transcript isoforms of more than one gene (as defined by a common gene symbol). To identify individually regulated mRNAs with DESeq2 v1.16.1, human read counts were normalized with the median ratio method before calculating mean fold-changes. *p*-values for changes in mRNA expression were obtained from a likelihood ratio test [114].

### Ribo-Seq

Sequencing of ribosome-protected fragments was performed as previously described [128]. Briefly, cells were washed once with ice-cold 1× PBS supplemented with 100 μg/ml cycloheximide (CHX) and harvested by scraping in ice-cold polysome lysis buffer (20 mM Tris-HCl pH 7.4, 10 mM MgCl_2_, 200 mM KCl, 1% [v/v] NP-40, 100 μg/ml CHX, 2 mM DTT, 5 U/ml RNasin) supplemented with 1× cOmplete EDTA-free Protease Inhibitor Cocktail (Roche). Lysates were rotated end-over-end for 10 min at 4 °C and cleared by centrifugation at 10,000*g* for 10 min at 4 °C. All samples were adjusted to the same A260. 10% of the lysate was saved as input. The lysates were subsequently digested with RNase I (240 U per A260; Thermo Fisher Scientific) for 5 min at 4 °C. Samples were resolved on a linear 17.5–50% (w/v) sucrose density gradient by ultracentrifugation for 2.5 h at 35,000 rpm. Fractions were eluted from the top of the gradient using a density gradient fractionation system (Brandel) coupled to a UA-6 UV/VIS detector (Teledyne Isco). Polysome profiles were obtained by measuring absorbance at 254 nm. During elution, ~300 μl fractions were collected every 14 s and immediately mixed with solution II (10 mM Tris-HCl pH 7.5, 350 mM NaCl, 10 mM EDTA, 1% [w/v] SDS, 7 M urea). RNA was purified from the cytoplasmic lysate (input) and from pooled monosomal fractions using phenol:chloroform:isamylalcohol (25:24:1; AppliChem) by phase separation. RNA from both input and ribosome-protected fragments (RPFs) was subjected to rRNA depletion using the Ribo-Zero Gold Kit (Illumina). Input RNA was randomly fragmented by alkaline hydrolysis at pH 10.0 for 12 min at 95 °C. Fragmented RNA and RPFs were size-selected (25–35 nts) on a 15% polyacrylamide TBE-urea gel. After end-repair with T4 PNK (NEB), 3 ng RNA per sample was used for library preparation using the NEXTflex Small RNA-Seq Kit v3 (Perkin Elmer). Samples were equimolarly pooled and sequenced on a NextSeq550 device (Illumina) with 75 bases single end.

After removal of adapters with the FASTX-toolkit (http://hannonlab.cshl.edu/fastx_toolkit/) and trimming of the four random nucleotides at the beginning and the end of the reads, alignment was performed with Bowtie v1.2.2 [107]. Reads that did not map to tRNA or rRNA were aligned to a human transcriptome (Gencode V27 as downloaded from the UCSC Genome Browser wgEncodeGencodeBasic27 table). In order to summarize reads at the gene level, only 25–35-nt-long reads that map to the annotated ORF of isoforms of one specific gene (as defined by a common gene symbol) were counted with an in-house-developed R script. Respective to the 5′end of the read, an offset of 12 nts upstream of the start codon and 15 nts upstream of the stop codon was assumed. In order to identify individually regulated mRNAs with DESeq2 v1.16.1, read counts were normalized with the median ratio method.

### Generation of DIG-labeled RNA probes by in vitro transcription or 3′end labeling

DIG-labeled RNA probes against exons 1 and 2 of rabbit β-globin (bex1/2) or human NCL were synthesized in vitro using the MEGAScript SP6/T7 Transcription Kit and DIG RNA labeling mix (Roche) according to the manufacturer’s instructions.

In total, 500 pmol of oligo d(T)_18_ was labeled with the DIG Oligonucleotide 3′-End Labeling Kit (Roche). Reactions were incubated at 37 °C for 45 min and stopped by the addition of 2 μl 0.2 M EDTA. All labeled oligonucleotides were immediately frozen at −80 °C.

### RNA gel electrophoresis and northern blotting

Detection of poly(A) RNA, *β-globin*, and *NCL* mRNA was performed as previously described [30, 57]. Briefly, 3–7 μg total RNA were mixed with 2× MOPS loading buffer (40 mM MOPS pH 7.0, 1 mM NaAc, 10 mM EDTA, 51.4% [v/v] formamide, 6.8% [v/v] formaldehyde, 7.1% [v/v] glycerol, 50 μg/ml ethidium bromide, bromophenol blue) and denatured for 10 min at 65 °C. RNA was resolved by size using 1.6% or 1.1% [w/v] agarose/2% [v/v] formaldehyde/1× MOPS (20 mM MOPS pH 7.0, 0.5 mM NaAc, 1 mM EDTA) gels. Loading and electrophoresis were examined under UV light and RNA was blotted O/N with 8x SSC buffer (1.2 M NaCl, 120 mM sodium citrate) onto Hybond-N^+^ nylon membranes (GE Healthcare). RNA was immobilized on membranes by UV crosslinking at 254 nm with 2× 120 mJ/cm^2^. Membranes were briefly rinsed in 2× SSC buffer and transferred to hybridization glass tubes.

For the detection of *β-globin* and *NCL* mRNA, prehybridization was performed with 10 ml of hybridization buffer (50% [v/v] formamide, 5× SSC, 0.1% [w/v] Ficoll 400, 0.1% [w/v] polyvinylpyrrolidone, 0.1% [w/v] BSA fraction V, 5 mM EDTA, 10 mM PIPES, 0.4 mg/ml yeast RNA, 1% [w/v] SDS) for 30 min at 55 °C. Then, 500 ng DIG-labeled β-globin or NCL probes were denatured for 5 min at 95 °C and added to prehybridized membranes. Hybridization was performed at 55 °C in a rotation oven O/N. Membranes were washed twice with pre-warmed 2× SSC/0.1% [w/v] SDS for 5 min at 65 °C and twice with pre-warmed 0.5× SSC/0.1% [w/v] SDS for 20 min at 65 °C. Membranes were briefly rinsed in 1× DIG wash buffer and blocked with 1× Northern Blot Blocking Solution (Roche) supplemented with 1× Maleic Acid buffer (10 mM maleic acid, 15 mM NaCl, pH 7.5) for 30 min at RT on a shaking platform. Alkaline phosphatase-coupled α-DIG antibodies (Roche) were added to the blocking solution at 1:5000 and membranes were further incubated for 30 min at RT. Membranes were washed 4× 15 min with 1× DIG wash buffer (10 mM maleic acid, 15 mM NaCl, 0.03% [v/v] Tween-20, pH 7.5). For detection, membranes were briefly rinsed in 1× DIG detection buffer (130 mM Tris-HCl pH 9.5, 100 mM NaCl) and incubated for 5 min with CDP-Star substrate (Perkin Elmer) diluted 1:10 in 1× DIG detection buffer. Membranes were wrapped in foil and exposed to X-ray films or developed with a Fusion FX chemiluminescence detector (Vilber).

For the detection of poly(A) RNA, prehybridization was performed with 5 ml of hybridization buffer for 30 min at 30 °C. A total of 500 pmol of DIG-labeled oligo d(T)_18_ probe was denatured for 5 min at 95 °C and added to prehybridized membranes. Hybridization was performed at 30 °C in a rotation oven O/N. Membranes were washed twice with pre-warmed 2× SSC/0.1% [w/v] SDS for 5 min at 30 °C and twice with 0.1× SSC/0.1% [w/v] SDS for 15 min at RT. Further processing of membranes was performed as described above.

### RT-qPCR

0.5–1 μg of on-column DNase digested total RNA was reverse transcribed with 100 U M-MLV Reverse Transcriptase (Promega) in the presence of 4 μM random hexamer primer (Thermo Fisher Scientific), 20 U RNasin RNase inhibitor (Promega), and 1 mM dNTPs (each) for 1 h at 37 °C. PCR reactions were assembled in 384-well plates using a 5-fold diluted cDNA reaction, 400 nM of each gene-specific primer and the PowerUp SYBR Green Master Mix (Thermo Fisher Scientific) in a final volume of 10 μl per well. Quantitative PCR was performed on a QuantStudio 5 system (Thermo Fisher Scientific). Triplicates were measured for every target/reference gene. To verify removal of genomic DNA and to avoid genomic signals, all measurements included negative controls where the reverse transcriptase was omitted from the cDNA reaction. To measure the efficiency of individual primer pairs, dilution series of one cDNA sample were prepared to generate a standard curve. Gene-specific primer sequences used for the detection of mRNAs were designed with the Universal Probe Library Assay Design Center (Roche), were synthesized by Eurofins Genomics and are listed in Additional file 12.

### Western blotting

For an appropriate separation of differentially sized proteins, 5–20% polyacrylamide gradient Tris-glycine gels were made using standard protocols. The samples to be analyzed were mixed with an appropriate volume of 5× or 2× SDS sample buffer and denatured for 10 min at 95 °C. Then, 15–30 μg of total protein was loaded per sample and electrophoresis was performed in 1× Tris-glycine SDS running buffer (25 mM Tris, 250 mM glycine, 0.1% [w/v] SDS) with 30 mA/gel. Proteins were transferred onto 0.2-μm pore-sized nitrocellulose membranes (Peqlab) in 1× Tris-glycine blotting buffer (20 mM Tris, 150 mM glycine, 20% [v/v] EtOH) at 90 V for 3 h at 4 °C by using a wet blotting device. Loading and blotting efficiency was monitored by Ponceau S staining of membranes after blotting. Following de-staining with TBS-T (50 mM Tris-HCl pH 7.5, 150 mM NaCl, 0.1% [v/v] Tween-20), membranes were blocked with 5% (w/v) milk/1× PBS/0.01% (w/v) sodium azide for 1 h at RT on a shaking platform. Membranes were incubated with primary antibodies diluted in 1× PBS/0.01% (w/v) sodium azide overnight at 4 °C on a shaking platform. Membranes were washed 5–6 times over a time period of 1 h with TBS-T before incubation with horseradish peroxidase (HRP)-coupled secondary antibodies (Jackson ImmunoResearch) in 1× PBS for 1 h at RT on a shaking platform. After 5–6 washes with TBS-T over a time period of 1 h, membranes were incubated with Western Lightning Plus ECL (Perkin Elmer) or Clarity / Clarity Max ECL Substrate (Bio-Rad) for 1 min, wrapped in foil and developed using X-ray films or with a Fusion FX chemiluminescence detector (Vilber).

### Antibodies

The following primary antibodies were used for western blot analysis: rabbit polyclonal anti-histone H3 K27 (1:1000, ab4729, Abcam), mouse monoclonal anti-FLAG M2 (1:1000, F3165, Sigma), rabbit monoclonal anti-NOT7 (1:1000, #86665, Cell Signaling), rabbit polyclonal anti-acetylated lysine (1:1000, #9441, Cell Signaling), mouse monoclonal anti-SBP (1:1000, sc-101595, Santa Cruz), rat monoclonal anti-tubulin (1:1000, ab6160, Abcam), rabbit polyclonal anti-histone H3 (1:1000, ab1791, Abcam), rabbit monoclonal anti-phosphorylated JNK (1:1000, #4668, Cell Signaling), rabbit polyclonal anti-JNK (1:1000, #9252, Cell Signaling), rabbit polyclonal anti-phosphorylated p38 (1:1000, #9211, Cell Signaling), rabbit polyclonal anti-p38 (1:1000, sc-535, Santa Cruz), mouse monoclonal anti-HA (1:1000, MMS-101P, Covance Innovative Antibodies), rabbit polyclonal anti-TOB1 (1:1000, 14915-1-AP, Proteintech), mouse monoclonal anti-PABP (1:1000, sc-32318, Santa Cruz), rabbit polyclonal anti-eIF4ENIF1 (1:1000, ab55881, Abcam), rabbit polyclonal anti-NOB1 (1:1000, ab201311, Abcam), rabbit polyclonal anti-ZNF385A (1:500, ab190111, Abcam), rabbit polyclonal anti-NOT1 (1:1000, 14276-1-AP, Proteintech), mouse monoclonal anti-HDAC1 (1:1000, sc-81598, Santa Cruz), rabbit monoclonal anti-NOT2 (1:1000, #34214, Cell Signaling), rabbit polyclonal anti-NOT10 (1:1000, 15938-1-AP, Proteintech), rabbit polyclonal anti-ERK1/2 (1:1000, #9102S, Cell Signaling), rabbit polyclonal anti-pERK1/2 (1:1000, #9101S, Cell Signaling), rabbit polyclonal anti-CPEB4 (1:1000, kindly provided by Raúl Méndez, IRB Barcelona), rabbit monoclonal anti-CAF1a (1:1000, kindly provided by Ann-Bin Shyu, University of Texas Medical School).

### Plasmid construction

The following plasmids have been described previously: pcDNA3-HA (p2003) [129], pTOPuro-mycStrep (p2484) [27], pcDNA3-HA-PP7cp-CAF1a-WT (p2742) [57], pcDNA3-HA-PP7cp (p2746) [130]. pCMV2-FLAG-CPEB1 (p3492), pRSETC-FLAG-mCPEB2 (p3908), pCMV7-3xFLAG-CPEB3 (p3469), and pCMV2-FLAG-CPEB4 (p3468) were kindly provided by Raúl Méndez (IRB Barcelona). A plasmid encoding hTOB1 (p3332) was kindly provided by Fabienne Mauxion / Bertrand Séraphin (IGBMC, Straßbourg). pSpCas9 (BB)-2A-GFP (p3511) was a gift from Feng Zhang (MIT, Addgene plasmid #48138).

To generate pcDNA3-HA-TOB1 (p3447), the coding sequence of TOB1 was amplified by PCR using G3674/G3675 and p3332 as template and inserted into p2003 via BamHI/XhoI.

pCMV2-FLAG-CPEB4 (p3468) corresponds to a splice variant of CPEB4 lacking 8 amino acids as compared to the full-length protein. To generate pCMV2-FLAG-CPEB4-complete ORF (p3491), p3468 was amplified by PCR using G3911/G3912 and used for Gibson assembly with annealed G3909/G3910. To generate pcDNA3-SBP-CPEB1 (p3564), pcDNA-SBP-mCPEB2 (p3924), pcDNA3-SBP-CPEB3 (p3565), and pcDNA3-SBP-CPEB4 (p3519), SBP coding sequence was first PCR amplified from p2484 with G3998/G4018 and inserted into p2003 via HindIII/EcoRV to yield pcDNA3-SBP (p3517). mCPEB2 coding sequence was PCR amplified with G5997/G5998 from p3908 and inserted into p3517 via XhoI/XbaI. CPEB3 coding sequence was excised from p3469 and inserted into p3517 via EcoRV. CPEB1 (G4339/G4340) and CPEB4 (G4019/G4020) coding sequences were PCR amplified from p3492 (CPEB1) or p3491 (CPEB4) and subsequently inserted into p3517 via EcoRV/XhoI. pcDNA3-SBP-Stop (p3546) was produced by site-directed mutagenesis of p3517 using G4230/G4231.

For pcDNA3-HA-PP7cp-CPEB1 (p3592), pcDNA3-HA-PP7cp-mCPEB2 (p3922), and pcDNA3-HA-PP7cp-CPEB4 (p3593), the CPEB1 (G4471/G4472), mCPEB2 (G5969/G5970), and CPEB4 (G4467/G4468) coding sequence was PCR amplified from p3564 (CPEB1), p3908 (mCPEB2), and p3519 (CPEB4) and inserted into p2742 via Gibson assembly following vector linearization with XhoI/BamHI. For pcDNA3-HA-PP7cp-CPEB3 (p3627), CPEB3 coding sequence was excised from p3565 with SalI/BamHI and inserted into p3554, which was derived from p2003 by site-directed mutagenesis using G4257/4258, via EcoRV following Klenow fill-in. From this intermediate, CPEB3 was subsequently excised and inserted into p2742 via BamHI/XhoI.

Scarless cloning of guide RNA sequence oligos was performed as previously described [131]. To produce pSpCas9(BB)-2A-GFP-*CPEB4*-guideA (p3646) and pSpCas9(BB)-2A-GFP-*CPEB4*-guideB (p3647), G4658/G4659 (guide A) or G4660/G4661 (guide B) were annealed and inserted into p3511 via BbsI sites.

Mutations, cloning boundaries, and coding sequences of all plasmids were verified by DNA sequencing (Eurofins Genomics). All DNA oligonucleotides used for cloning are listed in Additional file 12.

### Data analysis and statistical tests

Blots were processed and analyzed with ImageJ v2.0. High-throughput data were analyzed with R v3.6.3. Statistical analysis was performed using Microsoft Excel 2019 or Prism (GraphPad) v8.4.1. Statistical significance was calculated by performing a two-tailed, paired Student’s *t* test whenever an equal number of repeats was performed for every condition. When all values were calculated relative to a control treatment, control samples were set to 1 and a one-sample *t*-test was performed. Data are expressed as mean ± SD. A *p*-value < 0.05 was considered statistically significant.

## Supplementary Information

Additional file 1. Excel spreadsheet listing proteins identified by poly(A) RNA IC in DMSO-treated HeLa cells with LFQ values >10-fold above non-crosslinked background control.

Additional file 2. PDF file containing supplementary figures S1-S13 [59, 64, 99, 134].

Additional file 3. Excel spreadsheet listing proteins identified by poly(A) RNA IC in RMD-treated HeLa cells with LFQ values >10-fold above non-crosslinked background control.

Additional file 4. Excel spreadsheet listing proteins identified by poly(A) RNA IC with log_2_ fold-change ≥1 or ≤-1 (RMD/DMSO).

Additional file 5. Excel spreadsheet listing differentially expressed genes upon treatment with the class I-specific HDAC inhibitor RMD in HeLa cells.

Additional file 6. BED file containing CPEB4 PAR-CLIP peaks aligned to the hg19 version of the human genome.

Additional file 7. Excel spreadsheet listing accession numbers of HeLa CLIP datasets used in this study.

Additional file 8. Excel spreadsheet listing genes mapping to CPEB4 PAR-CLIP reads with diagnostic events.

Additional file 9. Excel spreadsheet listing mRNA half-lives determined in control (DMSO) and RMD-treated HeLa cells upon CPEB4 KD (S140, S181) or control KD (C2), or in untransfected cells.

Additional file 10. Excel spreadsheet listing ribosome densities determined from CPEB4 KO (CKO) and parental HeLa cells by Ribo-Seq analysis.

Additional file 11. Excel spreadsheet listing IEG mRNA half-lives and CPEB4 PAR-CLIP data.

Additional file 12. Excel spreadsheet listing DNA oligonucleotides used in this study.

Additional file 13. Excel spreadsheet containing source data and uncropped western / northern blots of Figs. 1B-E, 2A-C, 2E-F, 3A-C, 3E-G, 4A-F, 5A-F, 5H, 6A-B, 6D, 7A-F, S1A-C, S2A-C, S3B-D, S4, S5A-F, S6A-B, S8A-C, S10A-B, S11B-C, S12A-E, S13A-B.

Additional file 14. Review history.

## Data Availability

The datasets supporting the conclusions of this article are included within the article and its Additional files. Sequencing data supporting the conclusions of this article are available at the GEO database under the accession number GSE188694, which can be accessed via https://www.ncbi.nlm.nih.gov/geo/query/acc.cgi?acc=GSE188694 [132]. Proteomics data have been deposited at the ProteomeXchange Consortium via the PRIDE partner repository with the dataset identifier PXD029592, which can be accessed via https://www.ebi.ac.uk/pride/archive/projects/PXD029592 [133]. The universal pipeline for mapping of CLIP reads can be found at https://github.com/slebedeva/CLIP_mapping. The version of omniCLIP used in this study can be found at https://github.com/slebedeva/omniCLIP. The specific pipeline used to map CPEB4 PAR-CLIP reads, and the R code can be found in https://github.com/slebedeva/CPEB4_public [125]. Source data and uncropped western / northern blots of experiments shown in Figs. 1B–E, 2A–C, 2E–F, 3A–C, 3E–G, 4A–F, 5A–F, 5H, 6A–B, 6D, 7A–F, S1A–C, S2A–C, S3B–D, S4, S5A–F, S6A–B, S8A–C, S10A–B, S11B–C, S12A–E, S13A–B are provided in Additional file 13.
