# Supplementary material for: Control of immediate early gene expression by CPEB4-repressor complex-mediated mRNA degradation
Source: Genome Biol. 2022 Sep 12;23:193. doi: 10.1186/s13059-022-02760-5 (PMC9465963; doi:10.1186/s13059-022-02760-5)
Supplement: Supplementary file 2 — Additional file 2. PDF file containing supplementary figures S1-S13 [59, 64, 99, 134]. [file 13059_2022_2760_MOESM2_ESM.docx]

## Supplementary Figures

# **Control of immediate early gene expression by CPEB4-repressor complex-mediated mRNA degradation**

Fabian Poetz^1,2*^, Svetlana Lebedeva^3*^, Johanna Schott^1,2^, Doris Lindner^1,2^, Uwe Ohler^3,4^, and Georg Stoecklin^1,2^

^1^ Division of Biochemistry, Mannheim Institute for Innate Immunoscience (MI3) and Mannheim Cancer Center (MCC), Medical Faculty Mannheim, Heidelberg University, 68167 Mannheim, Germany

^2^ Center for Molecular Biology of Heidelberg University (ZMBH), 69120 Heidelberg, Germany

^3^ Berlin Institute for Molecular Systems Biology (BIMSB), Max Delbrück Center for Molecular Medicine, 10115 Berlin, Germany

^4^ Department of Biology, Humboldt Universität Berlin, 10099 Berlin, Germany

* these authors have contributed equally to this work


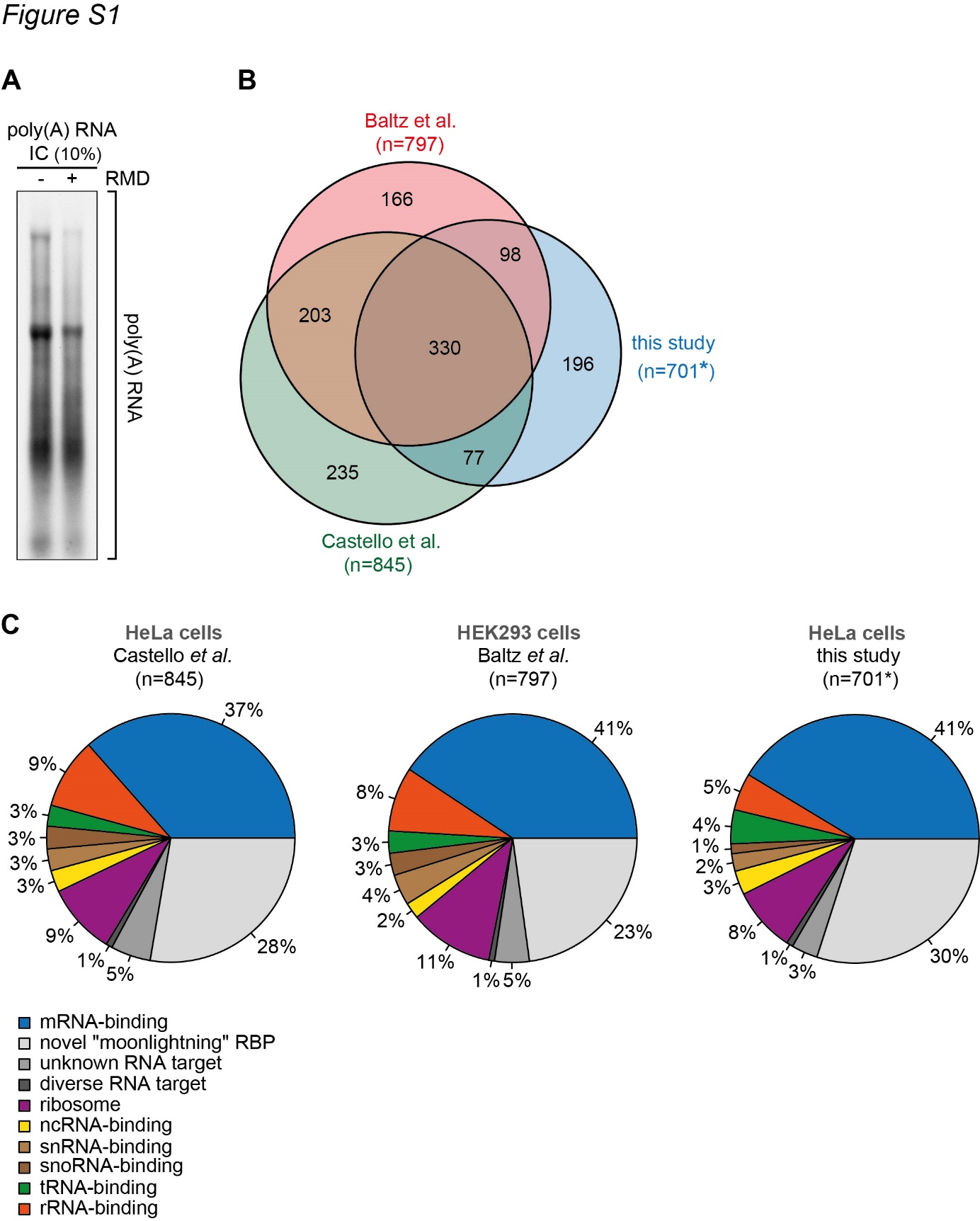


**Figure S1 | Poly(A) RNA interactome capture quality control (related to Figure 1).** (**A**) Prior to elution, RNA was recovered from 10% of the poly(A) RNA IC material. Integrity of poly(A) RNA was visualized by northern blot analysis using an oligo d(T)_18_ probe. (**B**) Venn diagram illustrating the comparison of proteins identified with confidence by poly(A) RNA IC in HeLa cells with the previously published studies of Castello *et al.*, 2012 [59] and Baltz *et al.*, 2012 [64]. This study identified 664 MaxQuant-assigned protein groups in two independent biological replicates with an at least 10-fold enrichment over non-crosslinked controls in the DMSO-treated condition. The 664 protein groups were deconvoluted into 701 unique gene symbols (marked with *). (**C**) Pie charts illustrating the abundance of individual RBP classes in each poly(A) RNA IC dataset from (B) according to the annotation used by Gerstberger et al., 2014 [134].


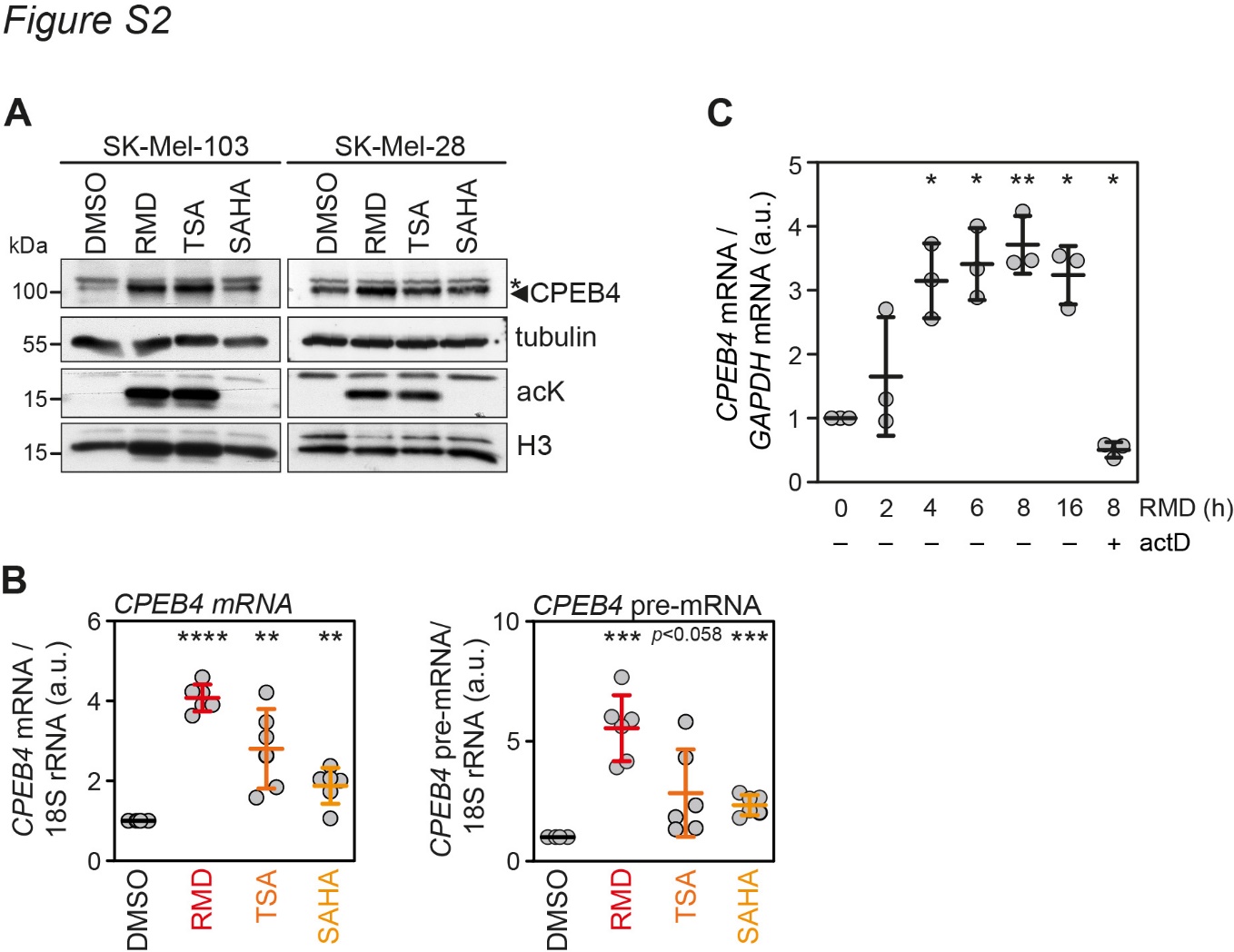


**Figure S2 | CPEB4 expression upon HDAC inhibition (related to Figure 2).** (**A**) Western blot analysis of CPEB4 expression in SK-Mel-103 and SK-Mel-28 cells upon treatment with 20 nM RMD, 500 nM TSA, 500 nM SAHA or an equal volume of solvent (DMSO) for 16 h. Data from one representative experiment are shown; the asterisk denotes a non-specific band. (**B**) *CPEB4* mRNA and pre-mRNA levels were measured by qRT-PCR in HeLa cells treated with 20 nM RMD, 500 nM Trichostatin A (TSA), 500 nM suberoylanilide hydroxamic acid (SAHA) or an equal volume of solvent (DMSO) for 16 h. 18S rRNA was used for normalization; data are presented as mean ± SD (n = 6). *p*-values were calculated using a two-tailed, one-sample t-test. (**C**) CPEB4 mRNA expression was examined by qRT-PCR following treatment of HeLa cells with 20 nM RMD at the indicated time points; in the last sample RMD was added together with 5 µg/ml actinomycin D over 8 h. GAPDH mRNA was used for normalization; data are presented as mean ± SD (n = 3). * *p* < 0.05, ** *p* < 0.01, *** *p* < 0.001, **** *p* < 0.0001.


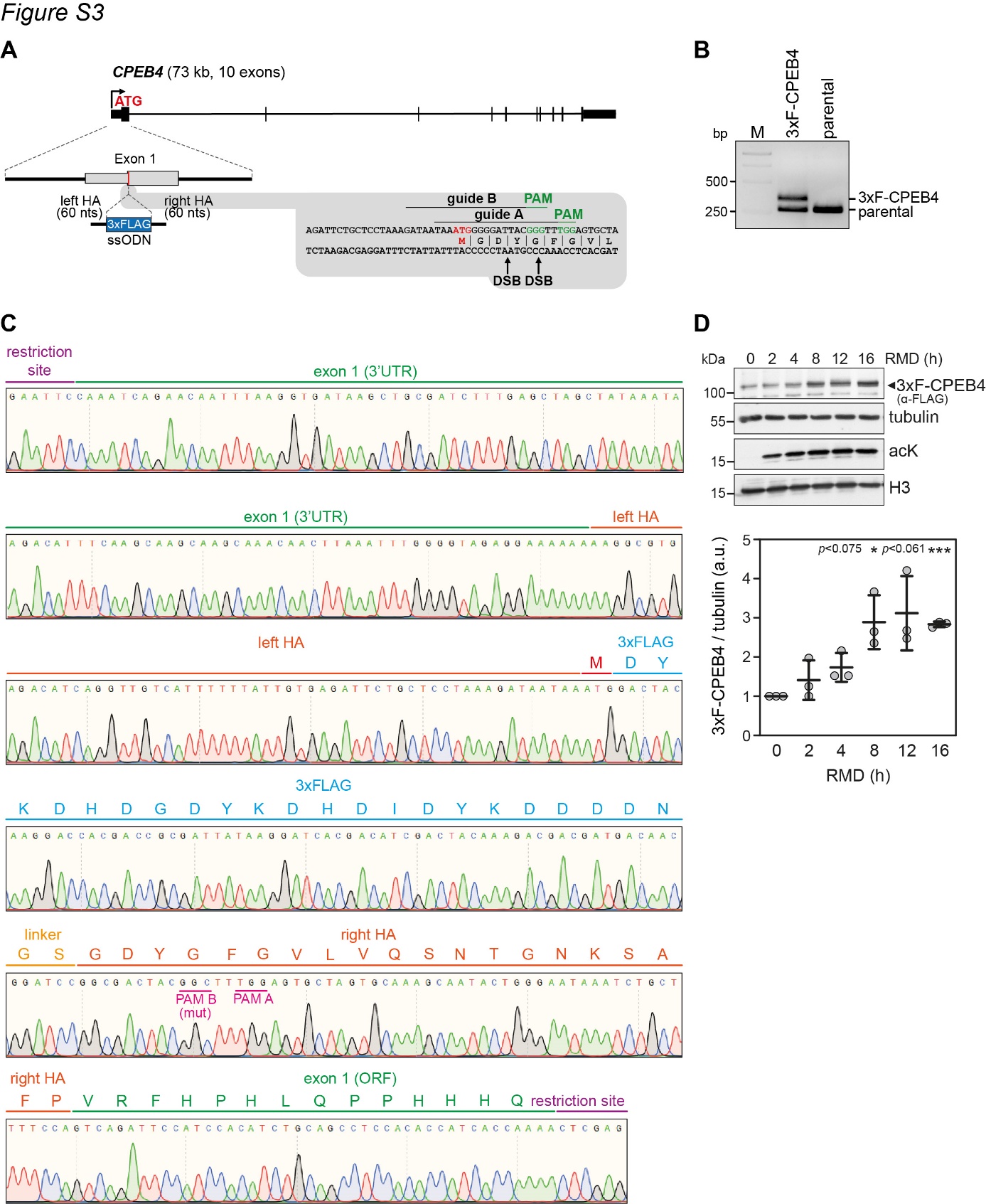


**Figure S3 | CRISPR/Cas9-mediated endogenous tagging of CPEB4 (related to Figure 2 and 4).** (**A**) Schematic illustration of the CRISPR/Cas9 strategy used to generate N-terminally tagged CPEB4 in HeLa cells. Integration of the 3xFLAG (3xF) sequence downstream of the ATG start codon was mediated by homology-directed repair using a single-stranded donor oligonucleotide and cleavage directed by two guide RNAs as indicated; DSB, double-strand break; PAM, protospacer adjacent motif; HA: homology arm. (**B**) Out-out PCR analysis of HeLa-3xF-CPEB4 clone #81 and parental HeLa cells. A region of the CPEB4 genomic locus spanning the edited site was PCR amplified. PCR products were resolved by 1% agarose gel electrophoresis and stained with Midori Green. The larger PCR product provides evidence for a monoallelic integration of the 3xFLAG sequence. (**C**) Sequencing chromatogram of the edited CPEB4 genomic locus containing the desired 3xFLAG insertion downstream of the ATG start codon in HeLa-3xF-CPEB4 clone #81. The larger PCR product from (B) was sub-cloned and subjected to Sanger sequencing. (**D**) HeLa-3xF-CPEB4 clone #81 cells were treated with 20 nM RMD for the indicated time periods, and induction of 3xF-CPEB4 expression was monitored by western blot analysis (upper panel). Dot plot shows quantification of 3xF-CPEB4 expression (lower panel). Tubulin was used for normalization. Data are presented as mean ± SD (n = 3). Indicated *p*-values were calculated using a two-tailed, one sample t-test. * *p*<0.05, *** *p*<0.001.


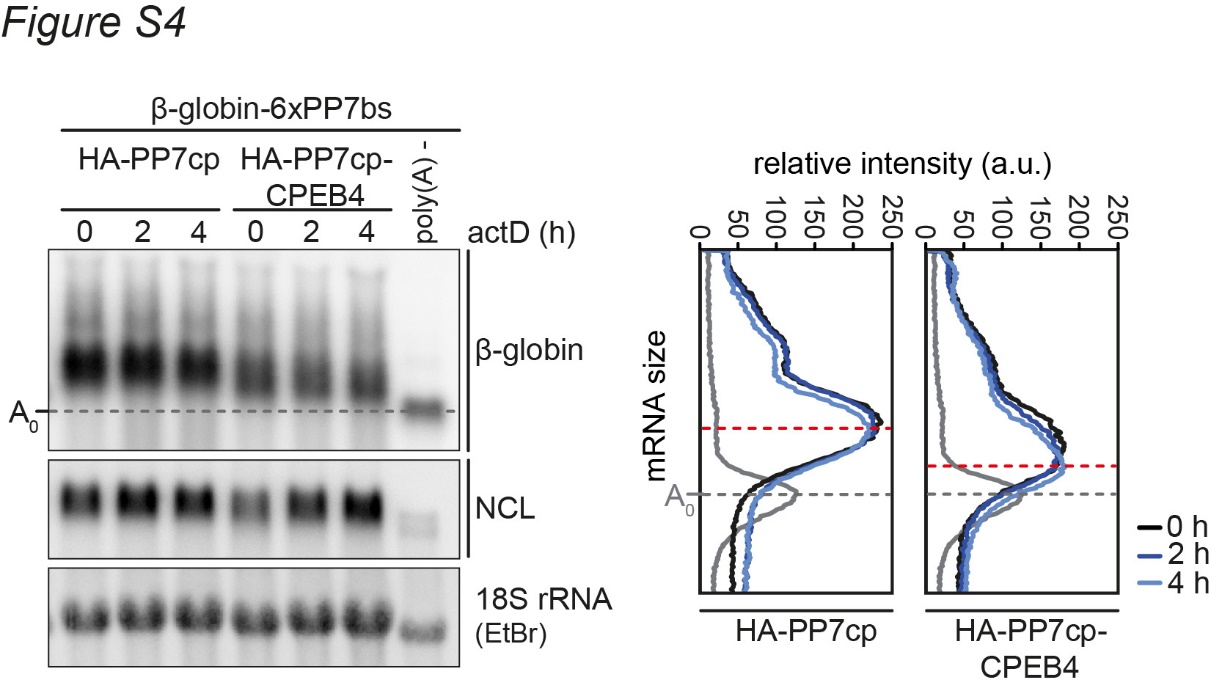


**Figure S4 | CPEB4-mediated deadenylation of *β-globin* mRNA in the tethering assay (related to Figure 3).** (**A**) CPEB4-mediated deadenylation of *β-globin* mRNA was analyzed in HeLa cells transiently transfected with HA-PP7cp or HA-PP7cp-CPEB4 together with a *β-globin* reporter mRNA containing 6 repeats of the PP7bs. Total RNA was extracted after 0, 2 or 4 hours of transcriptional shut-off with 5 μg/ml actinomycin D. *β-globin* reporter mRNA was visualized by northern blot analysis; 18S rRNA was visualized by ethidium bromide (EtBr) staining after blotting; *nucleolin* (NCL) mRNA serves as additional loading control (left side). To visualize poly(A) tail shortening of *β-globin* mRNA independently of its signal intensity, RNA quantities were adjusted to achieve comparable signal intensities in each lane. Deadenylation was visualized by densitometric analysis of the *β-globin* mRNA signal (right side). RNA digested with RNase H in presence of oligo-dT serves as a reference for fully deadenylated (poly(A) -) RNA. The signal intensity was plotted as a function of the poly(A) tail length and the peak maximum of the 4-h timepoint is indicated by a red dashed line.


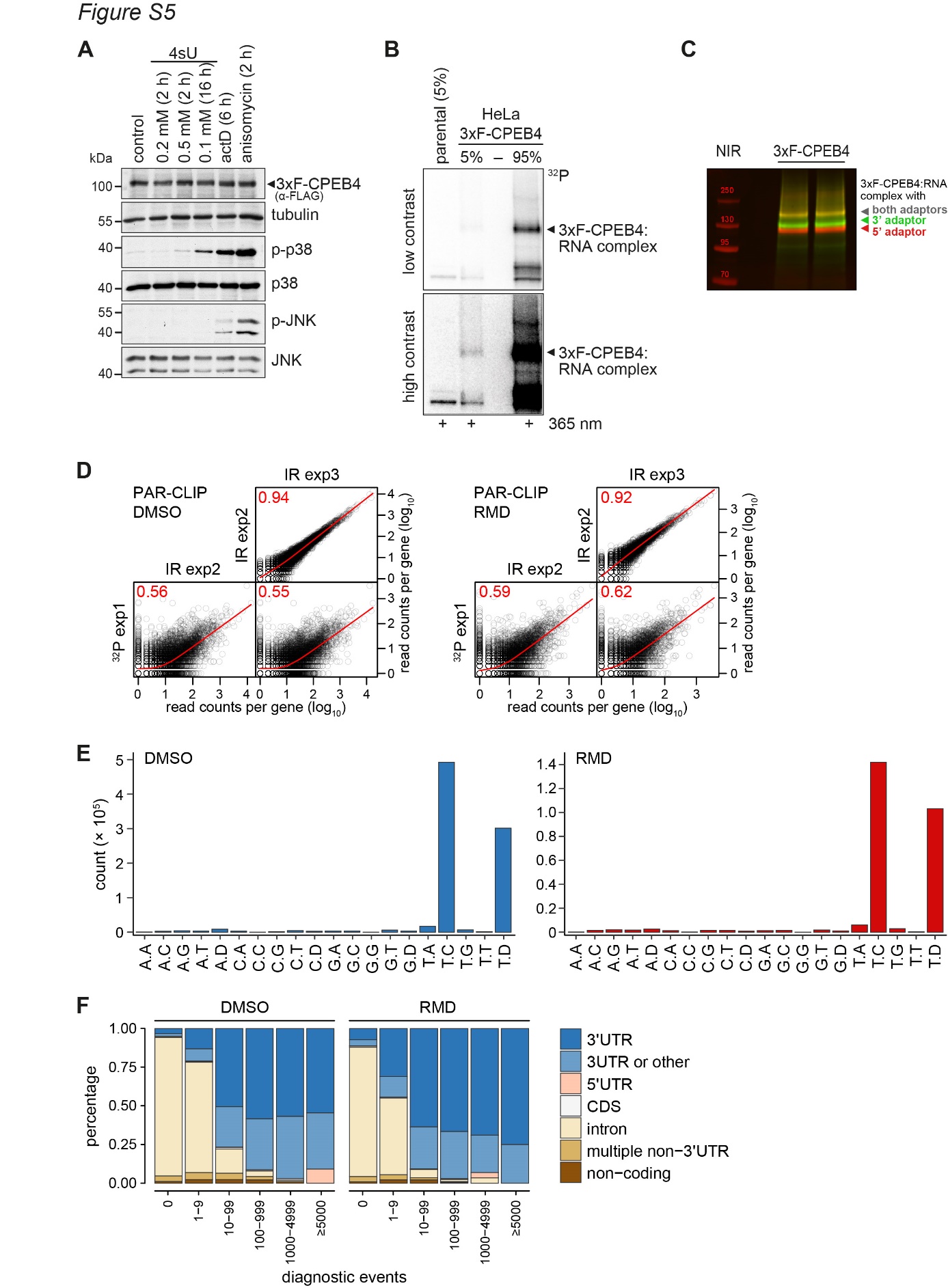


**Figure S5 | CPEB4 PAR-CLIP quality assessment (related to Figure 4).** (**A**) Western blot analysis of HeLa-3xF-CPEB4 cells treated with 4-thiouridine (4sU), 5 µg/ml actinomycin D (actD) or 1 µg/ml anisomycin as indicated. Phosphorylation of p38 MAPK at Thr183/Tyr185 and of JNK at Thr180/Tyr182 was monitored using phospho-specific antibodies; tubulin serves as loading control. (**B**) Autoradiograph showing CPEB4-bound RNA following FLAG IP-based enrichment of 3xF-CPEB4 and labeling with [γ-^32^P]-ATP. HeLa-3xF-CPEB4 cells were labeled with 200 µM 4sU for 2 h and UV-crosslinked at 365 nm. To assess background binding, the same FLAG IP was performed using parental HeLa cells. (**C**) Near-infrared scan showing CPEB4-bound RNA complexes following IP and adapter ligation. Complexes ligated to the 5ꞌ adapter only show in red color, to the 3ꞌ adapter only in green, and to both adapters in yellow; NIR, near-infrared protein ladder with size in kDa. (**D**) Scatterplots showing the correlation between CPEB4 PAR-CLIP read counts per gene for experiment 1 done with [γ-^32^P]-ATP labeling (^32^P) and experiments 2 and 3 done using infrared (IR). (**E**) Counts of mutations and deletions (designated as "N.D" where N = A, C, G or T) inside CPEB4 PAR-CLIP reads from DMSO- (left) and RMD-treated (right) cells. (**F**) The distribution of transcript categories containing CPEB4 binding sites was segregated according to the number of diagnostic events, comprising the sum of T deletions and T-to-C conversions. Category "3'UTR or other" indicates overlap of a 3ꞌUTR annotation and another category on the same strand. Category "multiple non-3ꞌUTR" includes any number of overlapping annotation categories except 3ꞌUTR.

**
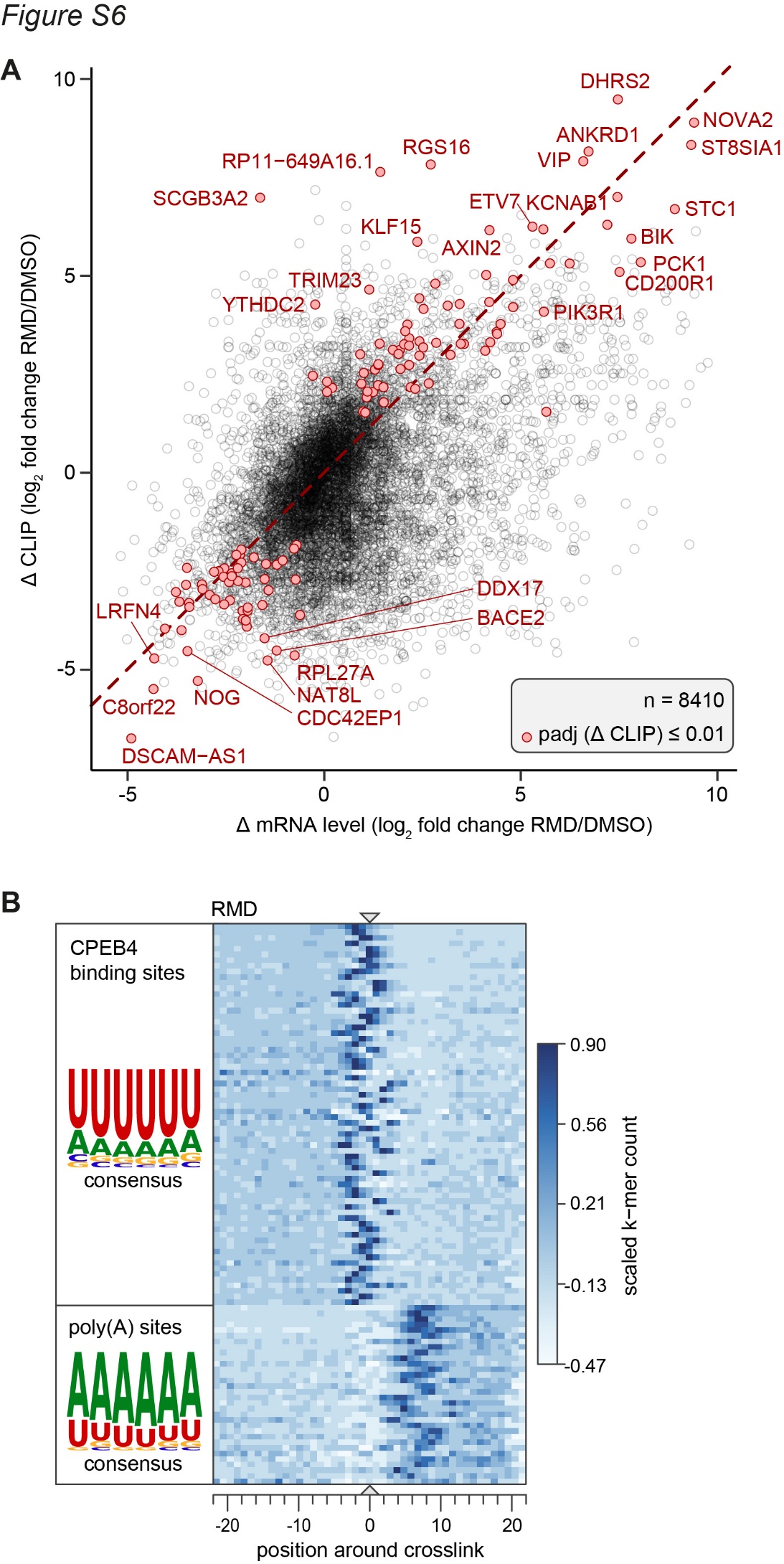
**

**Figure S6 | CPEB4 PAR-CLIP analysis (related to Figure 4). (A)** Scatterplot showing the log_2_-transformed fold change of CPEB4 PAR-CLIP reads per gene versus the fold change of mRNA levels between RMD- and DMSO-treated cells. Genes with significant changes in CLIP reads (padj < 0.01) are shown in red, and those that additionally show a log_2_ fold change > 4 are highlighted with gene symbols. (**B**) Heatmap showing scaled count of the 100 most abundant 6-mers in 50 nt windows around crosslink centers in RMD-treated HeLa-3xF-CPEB4 cells. Each line represents one 6-mer. Color intensity corresponds to scaled frequency of this 6-mer over all 3'UTR CPEB4 binding sites. The position refers to the center (nt 3) of the 6-mers. On the left side, 6-mers were segregated by position into 2 clusters using k-means clustering, and the positional weight matrix is shown for each cluster.

**
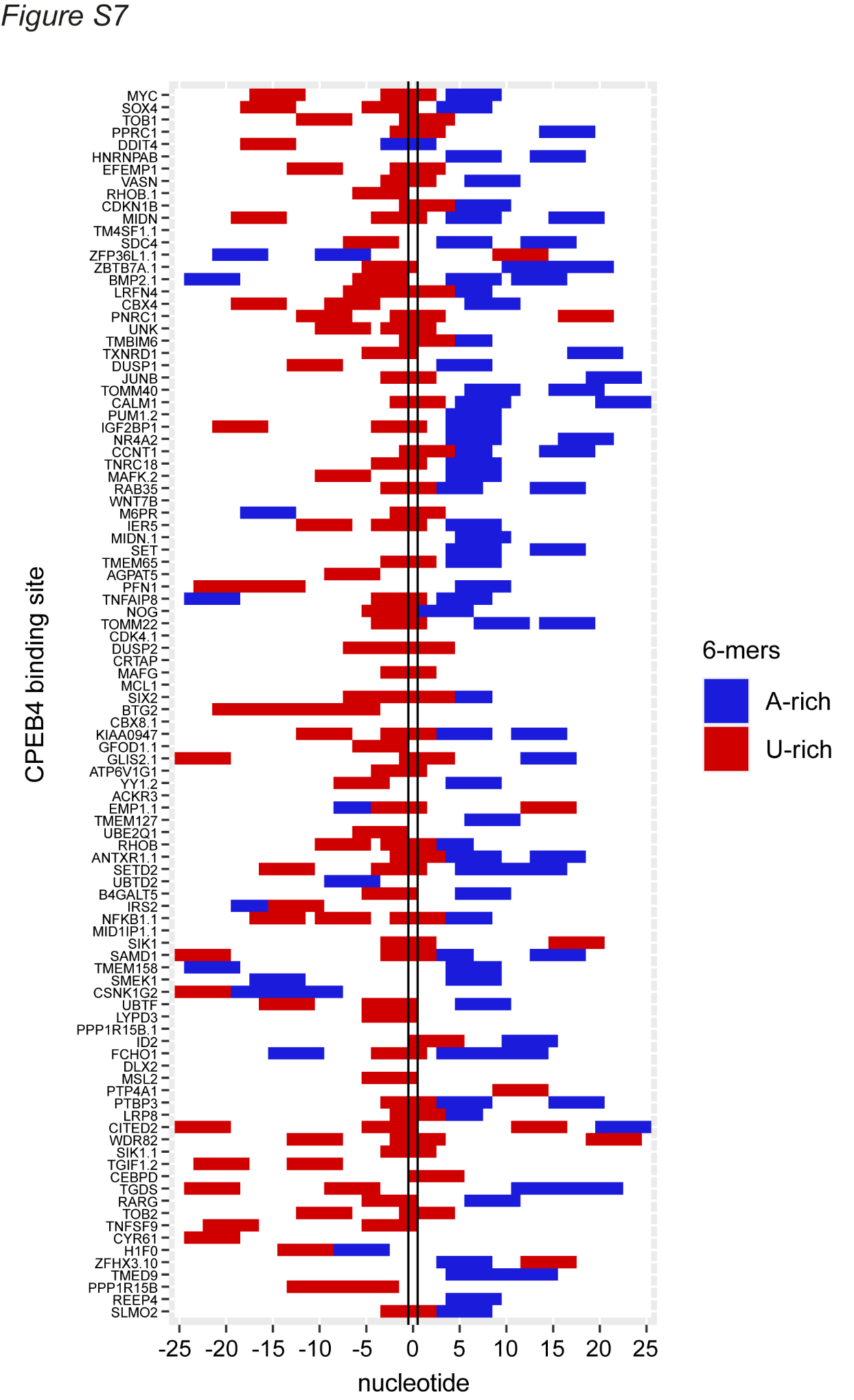
**

**Figure** **S7 | A-rich and U-rich hexamer locations in individual genes (related to Figure 4E).** The graph shows locations of top A-rich and U-rich 6-mers within +/-25 nt windows around crosslink centers in 3'UTRs for the top 100 CPEB4 target sites in the DMSO-treated condition. Each row represents a gene and rectangles represent enriched 6-mers. A-rich 6-mers are shown in blue and comprise 6-mers within the top 20 with at least 4 A nucleotides: AAUAAA, AUAAAA, UAAUAA, UAAAAA, AAAAAA, UAAAUA. U-rich 6-mers are shown in red and comprise 6-mers within the top 20 containing at least 4 U nucleotides: UUUGUA, UUUAUA, UUUUUA, UAUUUU, UUUUGU, UUUUAU, UUAUAU, AUUUUU, UUUUUG, UAUAUU, UUGUAU, CUUUUU. The crosslink center nucleotide is highlighted by two black lines. Target site IDs are shown on the left. These are identical to the respective gene name if the gene has only one binding site. Otherwise, gene names are numbered (SIK1, SIK1.1, etc). Target site IDs correspond to the binding site name in Additional file 6.

**
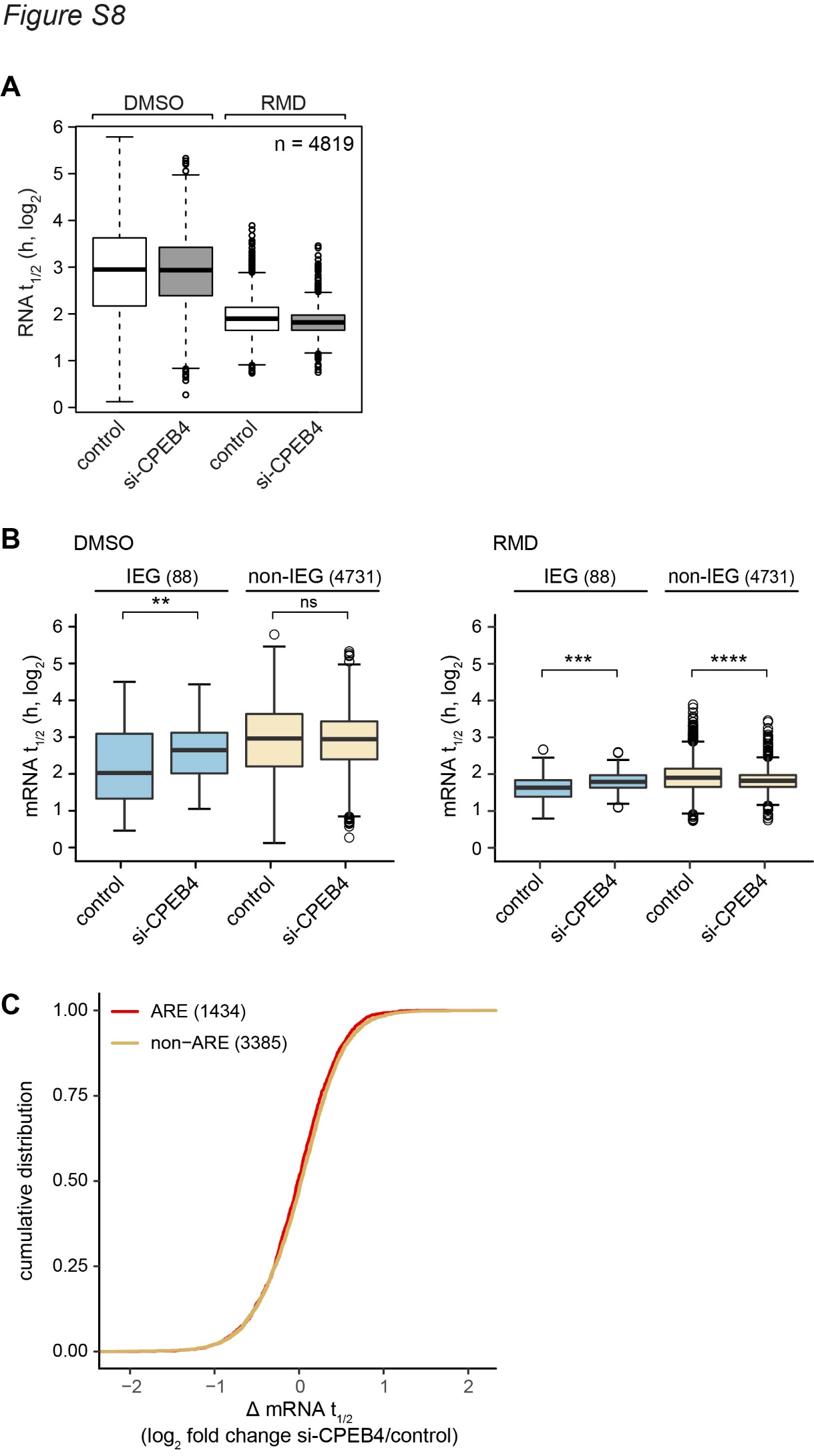
**

**Figure S8 | Effect of CPEB4 KD on mRNA turnover (related to Figure 5).** (**A**) Box plot showing the distribution of mRNA half-lives in control and CPEB4 KD HeLa cells treated either with 20 nM RMD or an equal volume of solvent (DMSO) for 16 h. Values are based on transcriptome-wide mRNA decay measurements by RNA-Seq analysis of actinomycin D chase experiments shown in Fig. 5C. (**B**) Distribution of half-lives of IEG and non-IEG mRNAs in control and CPEB4 KD HeLa cells treated with DMSO or RMD. *p*-values were calculated by two-sided Wilcoxon rank sum test; ns: *p* > 0.05, ** *p* ≤ 0.01, *** *p* ≤ 0.001, **** *p* ≤ 0.0001. (**C**) The cumulative distribution of log_2_-transformed changes in mRNA half-lives upon CPEB4 KD is shown for genes carrying an ARE in their 3ꞌUTR and all other genes.

**
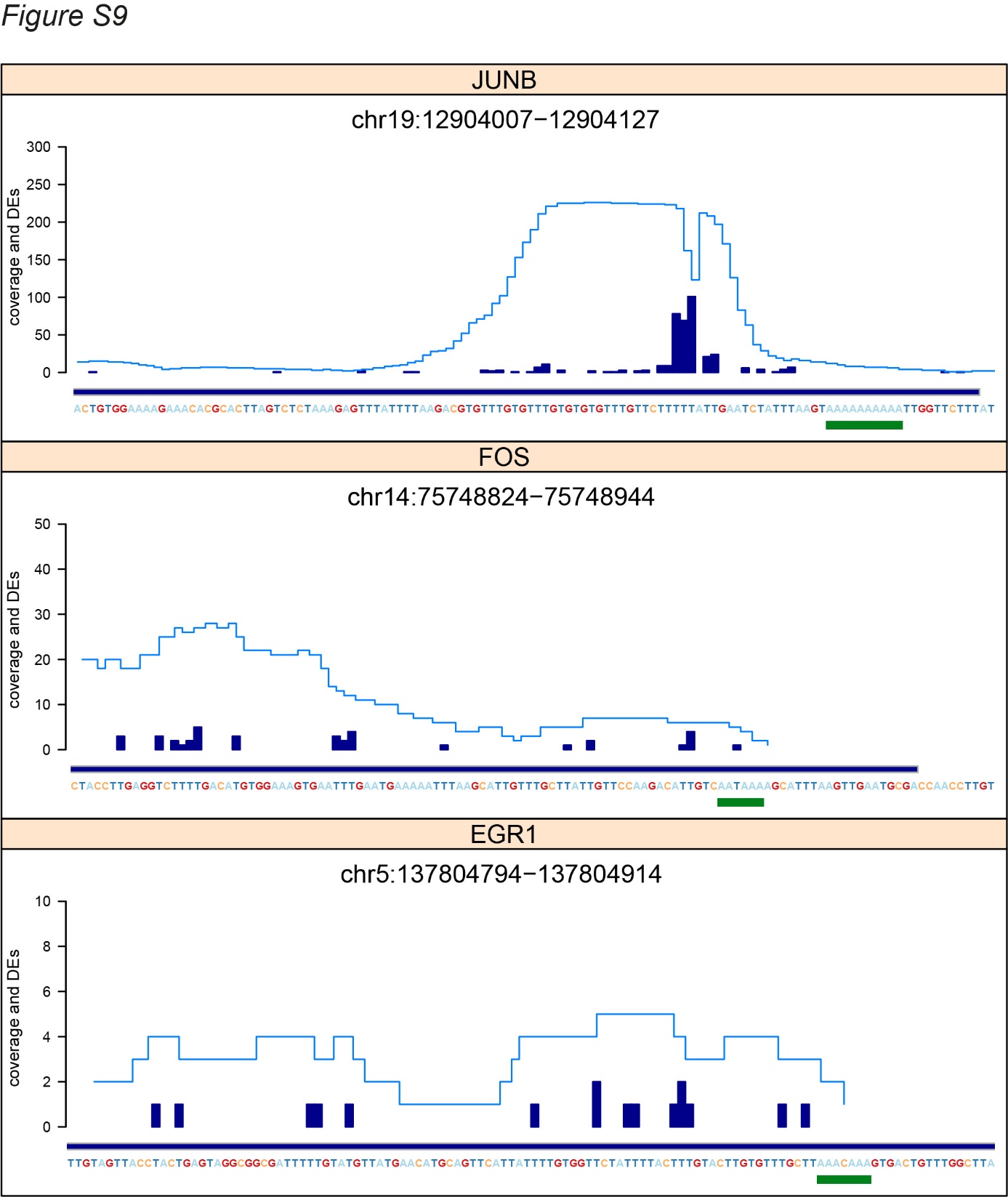
**

**Figure S9 | Nucleotide-level CPEB4 PAR-CLIP coverage and diagnostic events for three example IEGs (related to Figure 5G).** Nucleotide level zoom-in of the target sites for the genes, *JUNB*, *FOS* and *EGR1*. Coverage from a single replicate (^32^P) is shown as a light blue line, and diagnostic events (T-to-C conversions and T deletions) are represented as dark blue bars. Gene annotation is indicated by the dark blue line above the sequence; all genes are located on the plus strand. A-rich sequences are highlighted as green bars below the sequence. Top panel: *JUNB*, a strong CPEB4 target. A canonical AAUAAA hexamer is lacking, a stretch of A nucleotides is located downstream of the crosslink close to the annotated 3ꞌend. Middle panel: *FOS*, a medium CPEB4 target. CPEB4 coverage and diagnostic events are spread over a larger distance upstream of the annotated 3ꞌend, which carries the canonical AAUAAA poly(A) site. Bottom panel: *EGR1*, a weak target. The CPEB4 binding site is further upstream of the canonical poly(A) site, which is not seen on the panel.

**
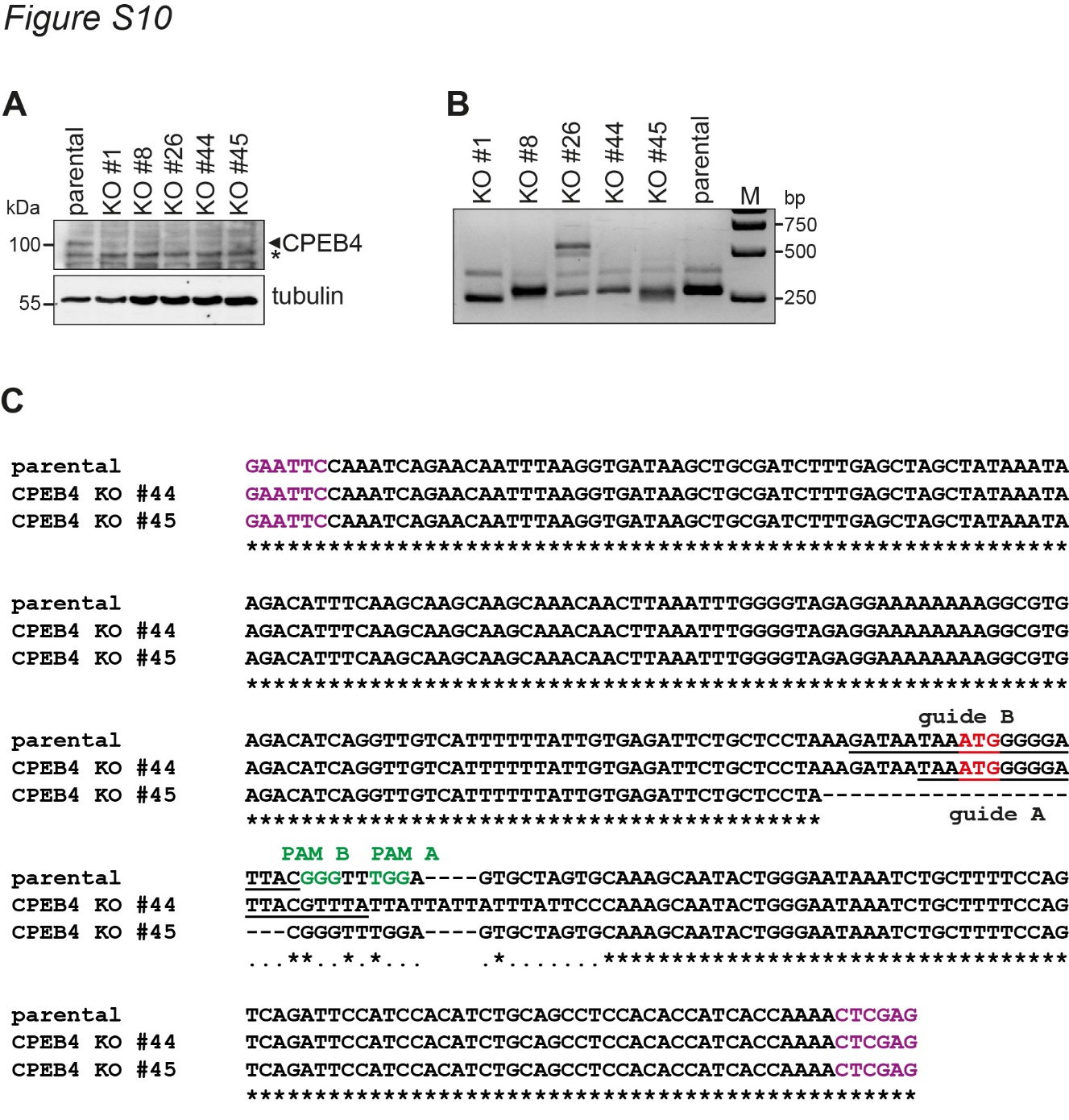
**

**Figure S10 | Verification of CRISPR/Cas9-mediated knock-out of *CPEB4* (related to Figure 5).** (**A**) Western blot analysis of CPEB4 protein expression in parental HeLa cells and different CPEB4 KO clones. The asterisk denotes a non-specific band. (**B**) Out-out PCR analysis of different CPEB4 KO clones and parental HeLa cells. A region of the *CPEB4* genomic locus spanning the edited site was PCR amplified. PCR products were resolved by 1% agarose gel electrophoresis and stained with Midori Green. (**C**) Sequence alignment of the edited *CPEB4* genomic locus derived from CPEB4 KO clones #44 and #45 with the unmodified sequence derived from parental HeLa cells. PCR products from (B) were sub-cloned and subjected to Sanger sequencing. The sequence of the two guide RNAs is underlined. The two PAMs, the ATG start codon and the restriction sites used for sub-cloning are highlighted in green, red, and purple, respectively. In clone #44, CRISPR/Cas9-mediated genome editing resulted in a short insertion of 4 nts downstream of the ATG start codon, whereas a larger sequence was deleted in clone #45.


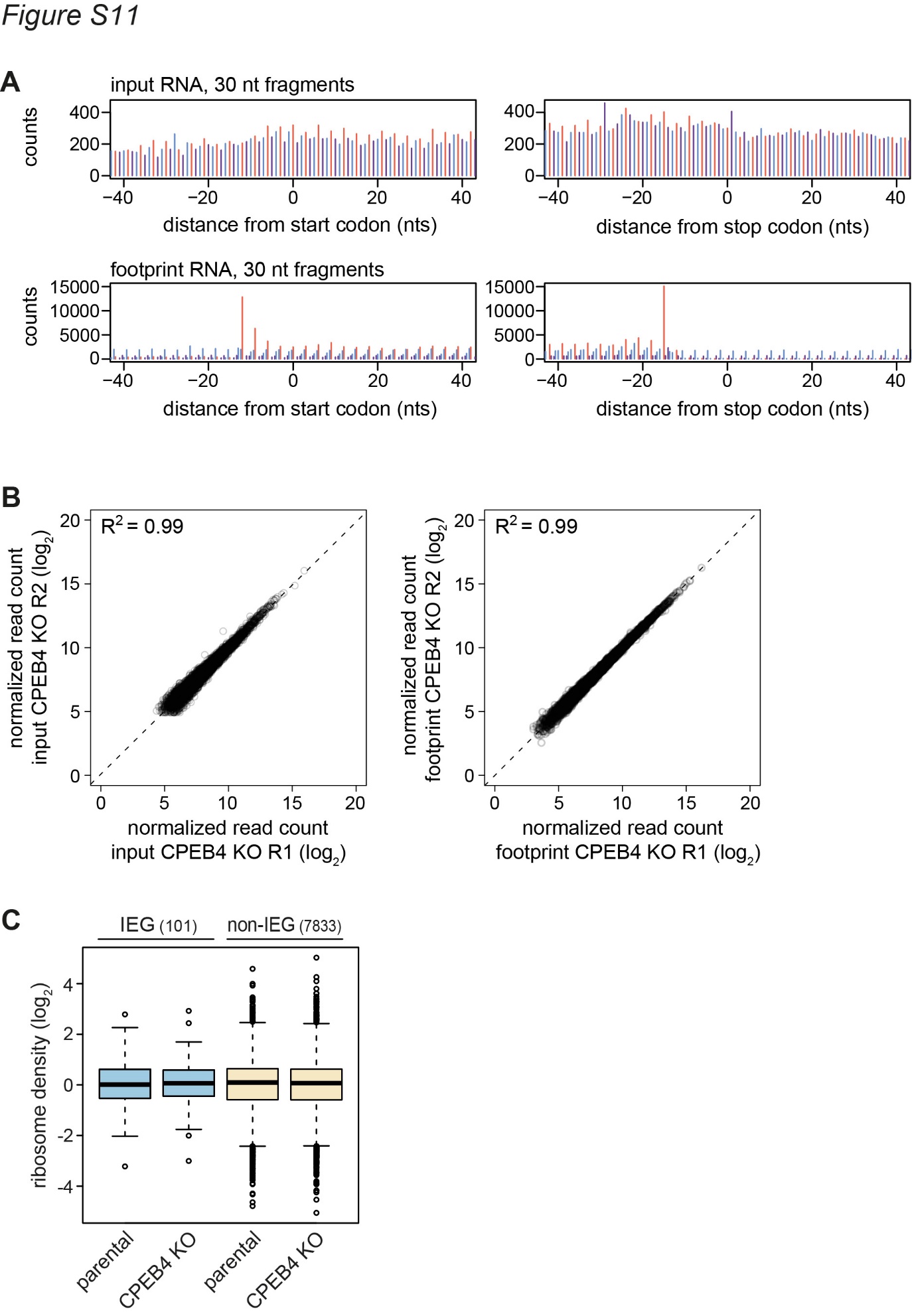


**Figure S11 | Ribo-Seq quality assessment and IEG analysis (related to Figure 5).** (**A**) Reads from one representative input RNA (top) and footprint sample (bottom) were aligned at the 5ꞌ end, and the distribution of all 30 nt long fragments is depicted according to their position relative to the start codon (left) and stop codon (right). (**B**) To assess reproducibility between repeat experiments, normalized read counts of two replicate experiments were plotted against each other. (**C**) Box plot depicting the distribution of ribosome densities of IEG and non-IEG mRNAs measured in two independent biological replicates using parental HeLa cells and CPEB4 KO clone #44.

**
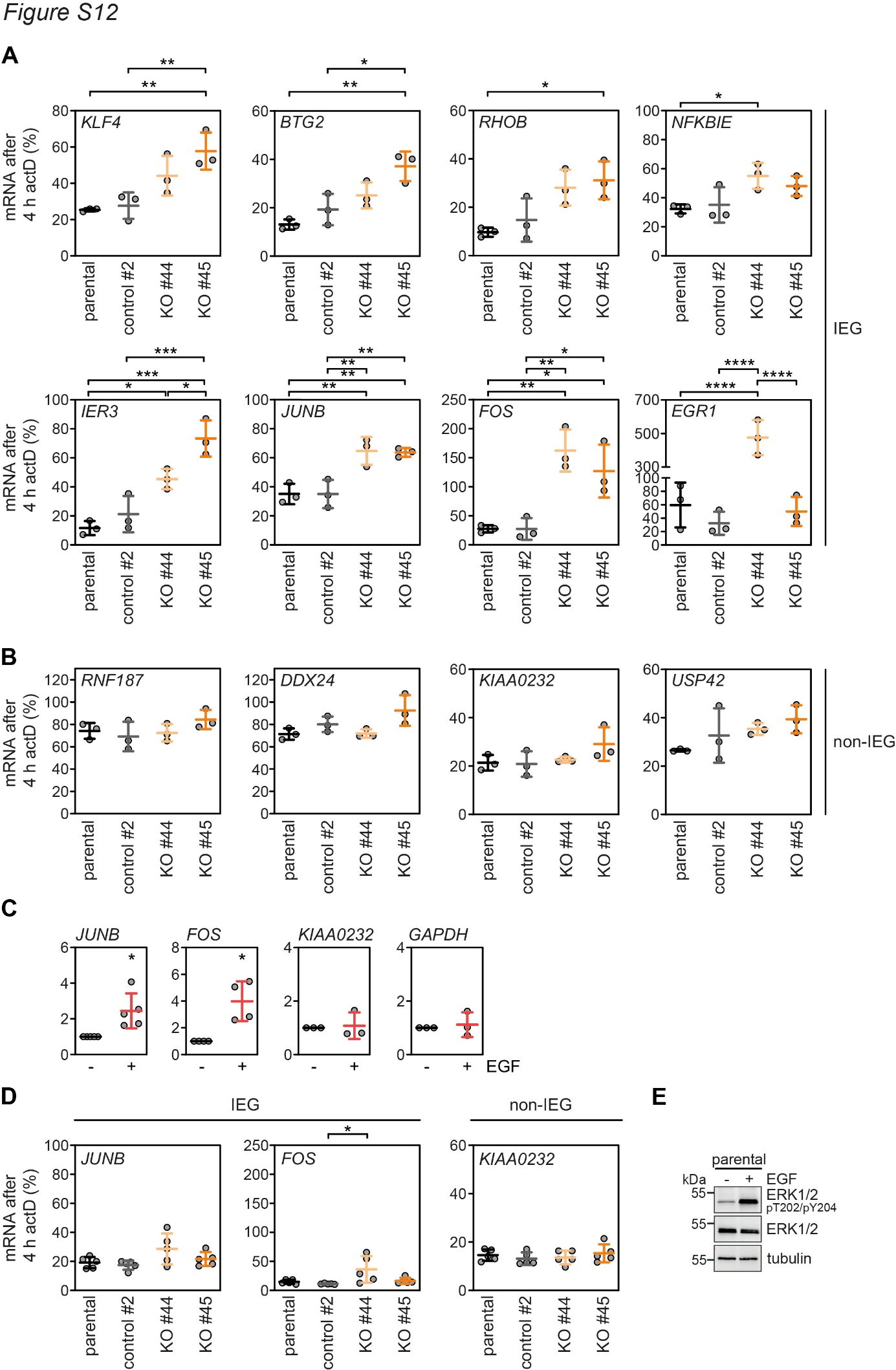
**

**Figure S12 | CPEB4-mediated IEG mRNA degradation (related to Figure 6).** (**A**) Dot plots depict the percentage of remaining mRNA after a 4-h transcriptional shut-off from mRNA decay measurements displayed in Fig. 6A and B. mRNA levels of different IEGs or (**B**) non-IEGs were measured by qRT-PCR. 18S rRNA was used for normalization. Data are presented as mean ± SD (n = 3). (**C**) Levels of IEG mRNAs *JUNB* and *FOS* as well as non-IEG mRNAs *KIAA0232* and *GAPDH* measured by qRT-PCR. HeLa cells were serum-starved for 24 h and stimulated with 20 ng/ml human EGF. (**D**) Dot plots depict the percentage of remaining mRNA after a 4-h transcriptional shut-off from mRNA decay measurements displayed in Fig. 6D. Indicated *p*-values were calculated using a one-way ANOVA test followed by Tukey’s multiple comparisons test. * *p*<0.05, ** *p*<0.01, *** *p*<0.001. (**E**) Western blot analysis of phosphorylated ERK1/2 (Thr202/Tyr204) upon EGF stimulation of HeLa cells as described in (C).


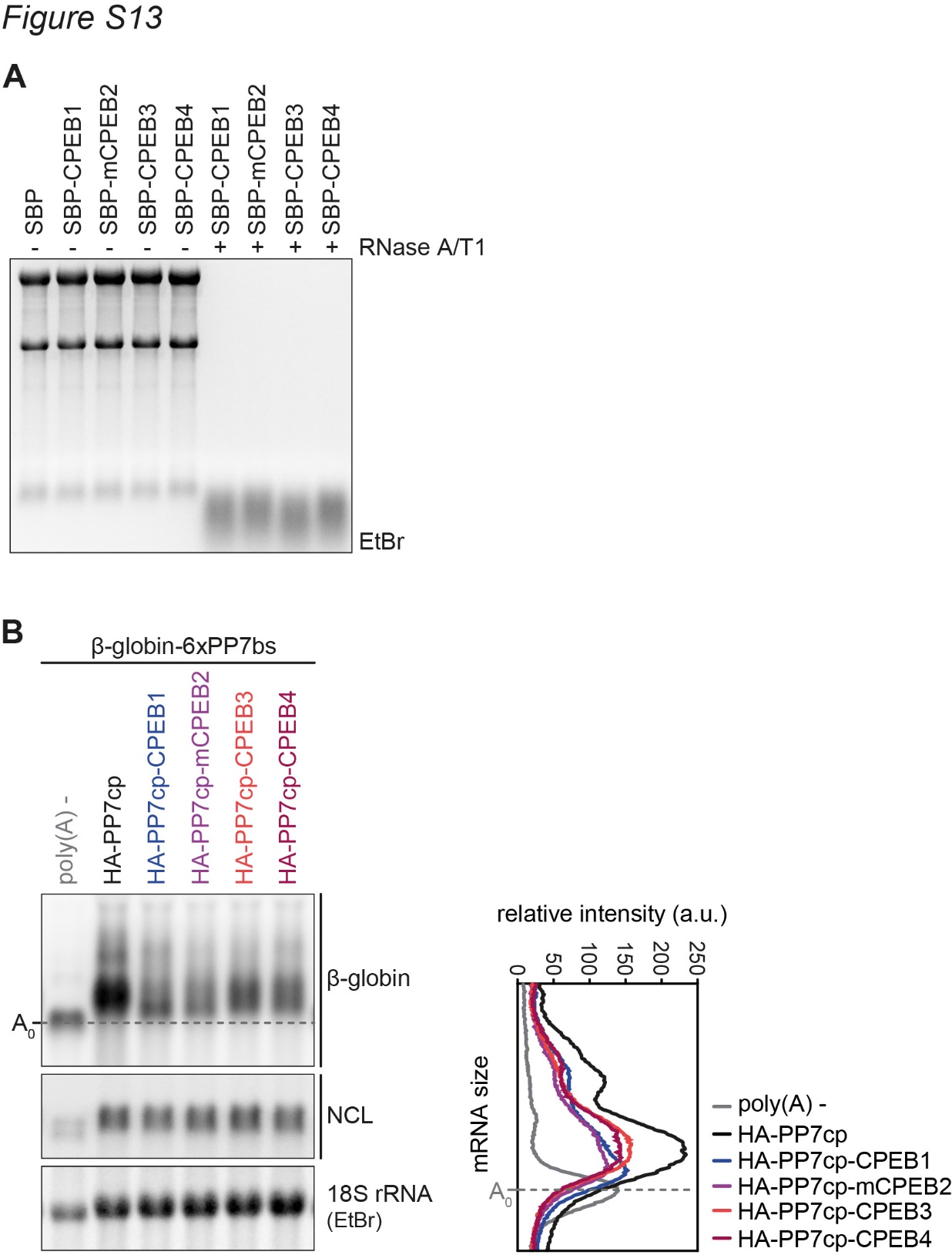


**Figure S13 | RNA quality control and CPEB-mediated deadenylation of *β-globin* mRNA at 0-hour timepoints (related to Figure 7).** (**A**) Total RNA was extracted from unbound material from IP samples shown in Fig. 7A, subjected to denaturing agarose gel electrophoresis, and stained with ethidium bromide (EtBr). (**B**) CPEB-mediated deadenylation of *β-globin* mRNA at steady state was analyzed in HeLa cells transiently transfected with HA-PP7cp or HA-PP7cp-tagged human CPEB1, CPEB3, CPEB4 or mouse mCPEB2 together with a *β-globin* reporter mRNA containing 6 repeats of the PP7bs. *β-globin* reporter mRNA from 0 h timepoints of the representative experiment displayed in Fig. 7B was visualized by northern blot analysis; 18S rRNA was visualized by ethidium bromide (EtBr) staining after blotting; *nucleolin* (NCL) mRNA serves as additional loading control (left side). Deadenylation was visualized by densitometric analysis of the *β-globin* mRNA signal (right side). RNA digested with RNase H in presence of oligo-dT serves as a reference for fully deadenylated (poly(A) -) RNA. The signal intensity was plotted as a function of the poly(A) tail length.

**
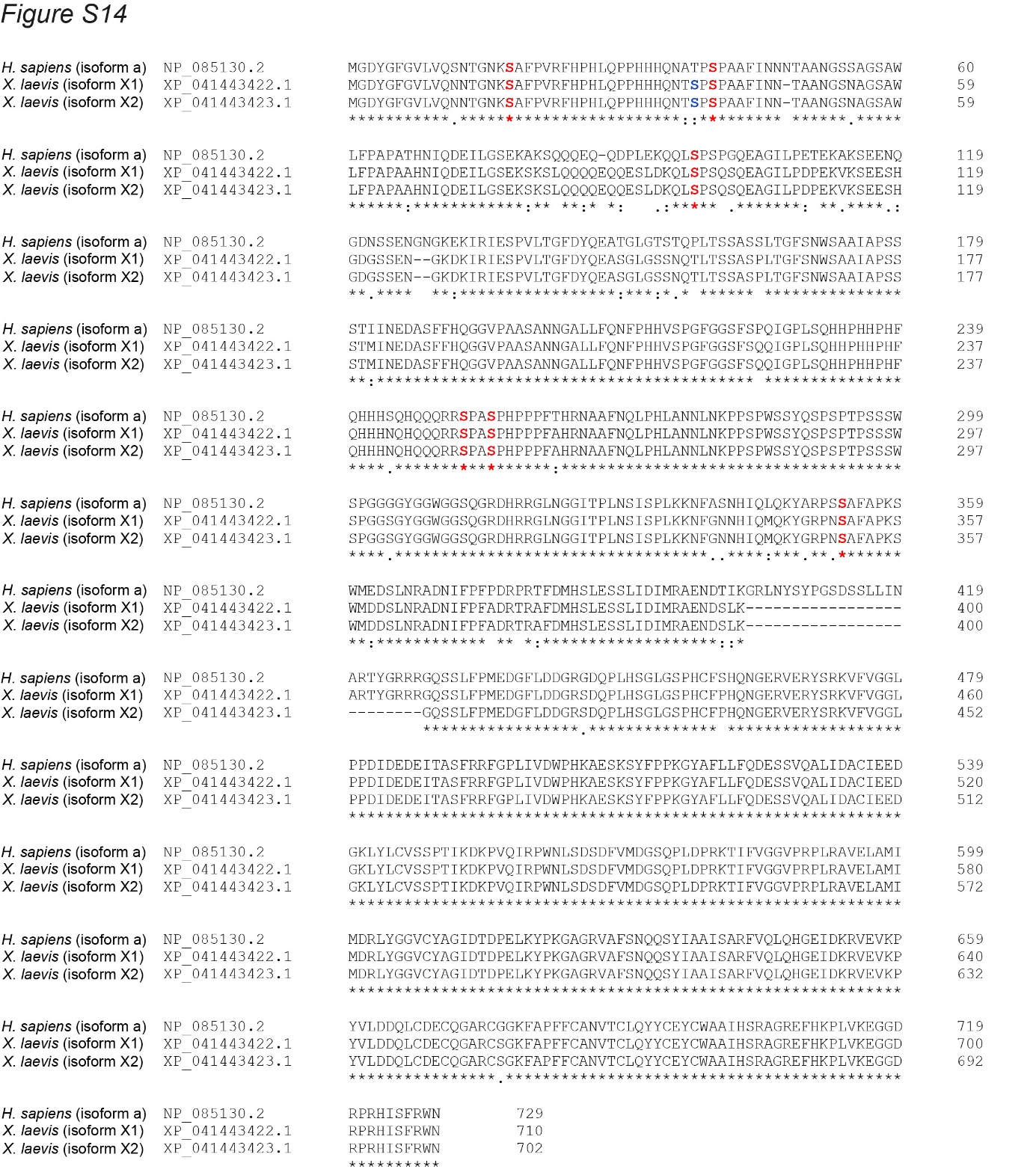
**

**Figure S14 | CPEB4 amino acid sequence conservation analysis.** The alignment covers CPEB4 homologues in human (*Homo sapiens*) and frog (*Xenopus laevis*). Conserved (S18, S40, S97, S250, S253, S351) and non-conserved (S38) ERK2-dependent phosphorylation sites identified by Guillén-Boixet *et al.* [99] are highlighted in red and blue, respectively.
