# Supplementary material for: Control of immediate early gene expression by CPEB4-repressor complex-mediated mRNA degradation
Source: Genome Biol. 2022 Sep 12;23:193. doi: 10.1186/s13059-022-02760-5 (PMC9465963; doi:10.1186/s13059-022-02760-5)
Supplement: Supplementary file 14 — Additional file 14. Review history. [file 13059_2022_2760_MOESM14_ESM.docx]

Review History

**First round of review**

**Reviewer 1**

**Are you able to assess all statistics in the manuscript, including the appropriateness of statistical tests used?** Yes, and I have assessed the statistics in my report.

**Comments to author:**

This is a very impressive series of highly difficult and challenging methods that the authors brought together to demonstrate how the RNA binding protein CPEB4, as a "RNA adaptor" mediates the AU-rich immediate early gene messenger RNAs. This work is based on the authors' earlier publication(s) (e.g. Sharma et al., 2016). These well-designed experiments use excellent statistics and have convincing conclusions. For example, these conclusions greatly advance the complex, yet exciting field of chromatin condensation/decondensation (e.g. HDACs) that links to posttranscription, and thus, I have no significant criticisms.

**Reviewer 2**

**Are you able to assess all statistics in the manuscript, including the appropriateness of statistical tests used?** Yes, and I have assessed the statistics in my report.

**Comments to author:**

The manuscript by Poetz, Lebedeva et al., describes the unexpected role of CPEB4 in mRNA turnover. The findings presented form part of what will likely become a wave of new research that questions long-standing ideas around the connection between the CPEB family, Cytoplasmic polyadenylation, translation and decay. As such, it holds an important place in redefining the role of this conserved family of RNA binding proteins in post-transcriptional gene regulation. Critically, the long-held view that CPEB proteins are involved in the stimulation of translation needs reassessing. If they stimulate translation, they do so in a temporal schema that is difficult to uncouple from their subsequent stimulation of mRNA decay.
The authors make a convincing case for CPEB4 in provoking the decay of immediate early genes (in HeLa cells) by recruiting the CCR4-NOT via TOB1. The data presented are sound and provide a compelling argument for a rethink in the current dogma. The comments provided below are therefore intended as prompts to improve the clarity of the presentation rather than a critique of the conclusions per se.

Text
line 124: hence-- reliable RBP from LOWER amounts of material. This comes out of the blue for the reader without further explanation (eg xx fold fewer cells than needed for traditional methods). As a reader, I was curious to know how much
line 222: -crosslinked nucleotides, in (THE ?) form of T-to-C transitions
line 279-81: sounds at first like it's a bit weak. Suggest adding a clause like 'indicating failsafe/redundant mechanisms for mRNA turnover' or something along those lines. CPEB4 was, afteral, identified in the RMD conditions suggesting its importance in that scenario. Does acetylation induced global mRNA turnover need a reference?

Figures

Figure 1:
-For the love of god and reader sanity ;) reorganise this figure to read left-to-right or up-and-down, not around in a meandering circle.
- Also, consider resizing the western blots throughout all figures to maintain the aspect ratio. The westerns in figures 2, 4 and 5 seem somewhat shrunken in comparison.
Figure 3:
-panel E: The northern bolt shows stimulated decay effectively but fails to convince with respect to deadenylation without normalisation for loading (eg same amount of signal in each lane to reduce the 'scatter' from overexposure) and a -poly(A) (eg RNase H treatment) control. Suggest a simple PCR based 3'-RACE could solve this issue. The poly(A)-distribution in PP7cp-CPEB4 holds evidence for deadenylation even before the transcriptional shutdown. It would be nice to show this more clearly. NCL is not mentioned in the text or figure legend.

Figure 4:
-Panel C: Suggest making it explicit on the figure that these are crosslink sites as opposed to the 6-mers in panel D. Why would there be more sites in DMSO? or is that a reflection of the number of mRNA? -in which case add the numbers. Eitherway, as you see, I'm not confident I understood this layout. Maybe it's because there's less RNA in which to detect the sites?
-Panel D: Maybe the GC rich 6-mers deserve a mention
-Panel E: It's slightly unsatisfying to have the plots un-tethered from each other so that it's not clear if the same genes that have the CPEB4 motif have the AAAAAA motif. An additional panel (F) that visually summarises the positioning of the motifs with respect to the end of the transcript would be good. Based on panel C, not all the sites are right at/overlapping the canonical AAUAAA motif which is normally within ~25 bases of the 3'UTR-polyA junction. This would be good to see, -given the ideas around the position of these elements and functionality in translational activation [ref 96].

Figure 5:
Panel G: Suggest putting in a zoomed-in section to highlight the 'codes' in these transcripts. A quick look in IGV failed to provide a satisfying picture that fits the premise of Fig 4 E for any given transcript and that should be discussed so that we are not left with ambiguous literature again regarding the precise positioning of these elements.

Figure 7:
Panel B: Given that the effect of CPEB4 stabilisation on protein expression is quite subtle (I couldn't convince myself by looking by eye, except maybe in the control at 4-hours), suggest plotting also the p-EGFR kinetics. There appears to be some variability in the activation between samples (eg timepoint 2 in CPEB4 samples seems less than control). A trace of this (or normalisation to it ?) may help. FOS is cropped too close to judge and should be cropped so that the full signal is included.
-Finally, the nail in the coffin of CPEB4 stimulating translation would be showing that the increased protein expression in siRNA samples is due to an increase in mRNA stability by qPCR or 3'-RACE. I think that would be an easy experiment and would be good to add.

**Authors Response**

**Point-by-point responses to the reviewers’ comments:**

Below please find a list of the major changes in the revised manuscript and our detailed response to the reviewers’ comments.

Summary of major changes:

1) During re-analysis of our PAR-CLIP data, a bug in the pipeline was discovered which causes some strand information loss during diagnostic event (DE) counting and affected a small fraction (~0.18%) of target genes. We now corrected this mistake in the pipeline. Since DE counts changed slightly, the respective figures (4B, 5A, 5E, 5F, S5F) have been updated. None of our main results or conclusions are affected by this change.

2) We changed the way to assign the CPEB4 crosslink center position. Previously, the center was assigned to a position with the highest count of T-to-C conversions among the three replicates. Since we rank the genes by the sum of all diagnostic events, the crosslink center is now assigned to the position with the highest count of both T-to-C conversions and T deletions summed up over all replicates. All figures that depend on the alignment at the crosslink position (4C-F, S6B), were updated in the revised manuscript. Again, none of our results or conclusions are affected by this change.

3) Based on the marginal difference between the timepoints with and without CPEB4 knock-down in Figure 7B of the initial manuscript, we decided to remove this unconvincing experiment. Instead of analyzing changes in IEG protein levels, we rather analyzed the impact of CPEB4 depletion on IEG mRNA stability following mitogenic stimulation with EGF, as suggested by reviewer #2. This analysis is now part of Figure 6 (panel C and D).

4) As mentioned above, we performed a comparative analysis of all four CPEBs and investigated their ability to interact with the CCR4-NOT complex and their ability to promote mRNA instability in a tethering approach (Figure 7). While our results show that all CPEBs associate with the CCR4-NOT complex, we observe interesting differences in the capacity of CPEBs to promote mRNA deadenylation and decay (with CPEB3 having the lowest activity), indicating that CPEBs assemble repressor complexes with different efficiency. This result fits very well to the finding by Duran Arqué et al. (parallel manuscript submitted to Genome Biol) that CPEB3, of all CPEBs, has the lowest tendency to form cytoplasmic foci.

5) We improved the analysis of β-globin reporter mRNA deadenylation by including a poly(A)-minus control as a reference, as suggested by reviewer #2.

All changes with respect to the previous version of our manuscript are highlighted in red. Source data and uncropped versions of western / northern blots of all Figures are supplied as an Excel spreadsheet (Additional File 12).

Data availability:
Sequencing data are available at the GEO database under the accession number GSE188694 with the reviewer token yrcdysuordoxdgd.
Proteomics data have been deposited at the ProteomeXchange Consortium via the PRIDE partner repository with the dataset identifier PXD029592. This dataset is accessible with the following account details: Username: reviewer_pxd029592@ebi.ac.uk; Password: Jfq23eSY.
The PAR-CLIP analysis pipeline in https://github.com/slebedeva/CPEB4_public will be made public upon acceptance. The content of the repository is available via <https://filetransfer.mdc-berlin.de/?u=KhK6kKGw&p=2w6nVjby&path=/supplementary_code_CPEB4_CLIP_analysis_revised.zip>.

*Reviewer #1:
This is a very impressive series of highly difficult and challenging methods that the authors brought together to demonstrate how the RNA binding protein CPEB4, as a "RNA adaptor" mediates the AU-rich immediate early gene messenger RNAs. This work is based on the authors' earlier publication(s) (e.g. Sharma et al., 2016). These well-designed experiments use excellent statistics and have convincing conclusions. For example, these conclusions greatly advance the complex, yet exciting field of chromatin condensation/decondensation (e.g. HDACs) that links to posttranscription, and thus, I have no significant criticisms.*

> Response: We highly appreciate the reviewer's encouraging assessment of our manuscript.

*Reviewer #2:
The manuscript by Poetz, Lebedeva et al., describes the unexpected role of CPEB4 in mRNA turnover. The findings presented form part of what will likely become a wave of new research that questions long-standing ideas around the connection between the CPEB family, cytoplasmic polyadenylation, translation and decay. As such, it holds an important place in redefining the role of this conserved family of RNA binding proteins in post-transcriptional gene regulation. Critically, the long-held view that CPEB proteins are involved in the stimulation of translation needs reassessing. If they stimulate translation, they do so in a temporal schema that is difficult to uncouple from their subsequent stimulation of mRNA decay.
The authors make a convincing case for CPEB4 in provoking the decay of immediate early genes (in HeLa cells) by recruiting the CCR4-NOT via TOB1. The data presented are sound and provide a compelling argument for a rethink in the current dogma. The comments provided below are therefore intended as prompts to improve the clarity of the presentation rather than a critique of the conclusions per se.*

> Response: We are pleased to see that the reviewer agrees with our conclusions. We have tried to improve the clarity of how the results are presented in our manuscript, as outlined below. To generalize our concept that CPEBs form repressive complexes, we added a comparative analysis of all four CPEB4 family members by investigating their ability to interact with the CCR4-NOT complex and to promote mRNA decay (new Figure 7).

Our new co-IP analysis demonstrates that all four CPEBs associate with the CCR4-NOT complex in an RNA-independent manner (Figure 7A). Using the tethering assay, we find that not only CPEB4 (Figure 3D–E), but also CPEB1 and CPEB2 can potently induce mRNA deadenylation and decay (Figure 7C-F). However, tethering of CPEB3 induced deadenylation only weakly, and did not lead to accelerated degradation of the reporter mRNA. Duran Arqué et al., in a manuscript submitted in parallel to Genome Biol, show that CPEB3 has the lowest propensity to form cytoplasmic foci among all four CPEBs. Thus, it is tempting to speculate that the activity of CPEBs within repressive complexes is connected to their phase separation behavior. We covered this aspect in our discussion (lines 392-397).

As a note: In Figure 7, we compared the human CPEB1/3/4 and mouse (m) CPEB2 proteins with each other. We tried several times to clone the human CPEB2 cDNA, but were not successful, and hence resorted to expressing the mouse CPEB2 protein from a cDNA provided by Raúl Méndez (IRB Barcelona). Since the human and mouse CPEB2 proteins share 98% sequence identity at the amino acid level, we believe that our conclusions also apply to human CPEB2. It is further worth mentioning that the expression levels of tagged CPEB1/2/3/4 from cDNA plasmids are vastly different, and that we adjusted the amounts of transfected plasmid in our tethering assays to achieve similar expression levels (Figure 7B).

*Text line 124: hence-- reliable RBP from LOWER amounts of material. This comes out of the blue for the reader without further explanation (eg xx fold fewer cells than needed for traditional methods). As a reader, I was curious to know how much.*

> Response: According to the methods paper published by Castello et al., 2013, poly(A) RNA interactome capture performed with UV-crosslinking requires ~2.8×108 cells per sample. Our revised protocol using more efficient formaldehyde crosslinking requires 7-8×106 cells per sample. This corresponds to a 35 to 40-fold lower amount of material that is required. This information has been included on lines 126/127.

*line 222: -crosslinked nucleotides, in (THE ?) form of T-to-C transitions*

> Response: We corrected the sentence as suggested (line 235).

*line 279-81: sounds at first like it's a bit weak. Suggest adding a clause like 'indicating failsafe/redundant mechanisms for mRNA turnover' or something along those lines. CPEB4 was, afterall, identified in the RMD conditions suggesting its importance in that scenario. Does acetylation induced global mRNA turnover need a reference?*

> Response: A reference for acetylation-induced mRNA turnover has been added (line 297).
CPEB4 was identified as an RBP with increased binding to poly(A) RNA upon RMD treatment (Figure 1) and contributes to the degradation of IEG mRNAs in the RMD-treated condition (Figure S8B). However, the contribution of CPEB4 to the turnover of this subgroup of mRNAs is rather weak (while still statistically significant) under these conditions, compared to the strong global mRNA destabilization that we observe upon HDAC inhibition. Attributing a strong impact of CPEB4 on IEG mRNA degradation under conditions of hyperacetylation would not be supported by our data.

*Figure 1:
For the love of god and reader sanity ;) reorganise this figure to read left-to-right or up-and-down, not around in a meandering circle.*

> Response: We changed the way the panels are arranged in this figure and pray that our reviewer keep his/her wit and sanity for many years to come.

*Also, consider resizing the western blots throughout all figures to maintain the aspect ratio. The westerns in figures 2, 4 and 5 seem somewhat shrunken in comparison.*

> Response: The western blots of all main figures have been resized accordingly.

*Figure 3:
Panel E: The northern bolt shows stimulated decay effectively but fails to convince with respect to deadenylation without normalisation for loading (eg same amount of signal in each lane to reduce the 'scatter' from overexposure) and a -poly(A) (eg RNase H treatment) control. Suggest a simple PCR based 3'-RACE could solve this issue. The poly(A)-distribution in PP7cp-CPEB4 holds evidence for deadenylation even before the transcriptional shutdown. It would be nice to show this more clearly. NCL is not mentioned in the text or figure legend.*

> Response: To analyze deadenylation independently of the reporter mRNA signal intensity, we adjusted the loading of the samples to achieve similar signals in every lane (Figure S4). Normalized loading is evident from almost identical signal intensities of the reporter mRNA and increasing levels of NCL and 18S rRNA in the 2- and 4-hour timepoints. Densitometric analysis clearly demonstrates accelerated deadenylation of the reporter mRNA upon tethering of CPEB4 as compared to HA-PP7cp alone. A poly(A)- control, obtained by RNase H treatment in presence of oligo-dT, was added as an internal reference.

To compare the polyadenylation status of the reporter mRNA upon expression of different CPEB family members at the 0-h timepoint, we loaded these samples next to each other, again including a poly(A)- control as a reference (Figure S13B). Compared to CPEB3 and CPEB4, tethering of CPEB1 and mouse CPEB2 led to pronounced deadenylation that is evident already at the 0-hour timepoint (Figure S13B), indicating different capacities to induce poly(A) tail shortening among the CPEB family members. Although CPEB4 is a potent inducer of mRNA deadenylation and decay (Figure 3E-G, Figure S4), deadenylation of the reporter mRNA at the 0-hour timepoint was barely visible.
Apart from the 18S rRNA, NCL serves as an additional loading control for all northern blots. This information was added to all figure legends.

*Figure 4:
Panel C: Suggest making it explicit on the figure that these are crosslink sites as opposed to the 6-mers in panel D. Why would there be more sites in DMSO? or is that a reflection of the number of mRNA? -in which case add the numbers. Eitherway, as you see, I'm not confident I understood this layout. Maybe it's because there's less RNA in which to detect the sites?*

> Response: There are several reasons why the coverage would look different for the RMD and DMSO conditions:
1. The number of binding sites plotted for DMSO vs. RMD may be different. Indeed, the DMSO sequencing libraries were deeper (the deepest DMSO replicate comprised 1.39 million uniquely mapped reads, and the deepest RMD replicate about 0.66 million) - hence more sites were called in the DMSO condition.
2. The size of the called clusters may be different: if one library is deeper - which is the case for the DMSO condition - there would be, on average, a longer area of binding called per site by omniCLIP. Indeed, the average length of the DMSO sites in 3’UTRs is around 43 nts, and of the RMD sites around 34 nts. In consequence, there will be more coverage for longer sites (e.g. 100 sites of length 100 have two times more coverage than 100 sites of size 50).
3. The size of the 3’UTRs may be different: if the 3’UTRs targeted by CPEB4 are different in size for the DMSO and RMD condition, and each 3’UTR is divided into 100 bins, the coverage profile will look different since one bin of a shorter 3’UTR accounts for a higher relative coverage.

To account for these three possible biases, we altered our analysis pipeline in the following way: 1. We plotted the same number of sites (3500 for each condition). 2. We plotted the coverage of 50 nt windows around the top crosslink position instead of the original sites to account for differences in size. 3. We only plot coverage on those 3’UTRs which are common to both conditions.

The result of the adjusted analysis is depicted in Figure 4C, showing that the coverage along the length of 3'UTRs is virtually identical between the DMSO and RMD condition.
We thank Bora Uyar, the author of the RCAS package, for help with the adjusted analysis.

*Panel D: Maybe the GC rich 6-mers deserve a mention*

> Response: In our initial analysis, the k-mer counts of all CPEB4 sites were normalized to the background k-mer counts in 3’UTRs. Since GC-rich 6-mers are very rare in both 3’UTRs and CPEB4 CLIP sites, their ratio is sensitive to stochastic variability. Again, possibly due to the deeper library size of the DMSO samples, there might have been more low confidence CPEB4 sites called in the DMSO condition, contributing to the GC-rich 6-mer counts. We now repeated this analysis by restricting the 6-mer counts to CPEB4 sites located in 3'UTRs only (as opposed to all CPEB4 sites, as in the original submission). The result of the new analysis is shown in Figure 4D. While GC-rich 6-mers are no longer enriched in the DMSO condition, the main U-rich CPEB4 sites and downstream A-rich motifs were identified in nearly identical order by the new analysis. We would like to thank the reviewer for this comment since it prompted us to substantially improve our analysis. As a consequence of the new analysis, the following graphs had to be replaced, without affecting any of our main conclusions: Figure 4D, 4E and S6B.

*Panel E: It's slightly unsatisfying to have the plots un-tethered from each other so that it's not clear if the same genes that have the CPEB4 motif have the AAAAAA motif. An additional panel (F) that visually summarizes the positioning of the motifs with respect to the end of the transcript would be good. Based on panel C, not all the sites are right at/overlapping the canonical AAUAAA motif which is normally within ~25 bases of the 3'UTR-polyA junction. This would be good to see, -given the ideas around the position of these elements and functionality in translational activation [ref 96].*

> Response: There is a misunderstanding behind this comment. Figure 4E (and S6B) does not show motifs on a per gene basis, but the counts (or density) of the 100 most abundant 6-mer motifs (one motif in each row) in the CPEB4 CLIP analysis. Hence, this heat map does not allow to combine positional information of U-rich and A-rich motifs that occur in the same 3'UTR. To do so, we visualized for the top 100 target DMSO binding sites the positions of the A-rich and U-rich 6-mers in a window of +/- 25 nts relative to the top crosslink site (Figure S7). In this analysis, A-rich 6-mers (green boxes) are defined as any of the top 20 6-mers which have 4 or more A’s: "AAUAAA" "AUAAAA" "UAAUAA" "UAAAAA" "AAAAAA" "UAAAUA". Similarly, U-rich 6-mers (red boxes) are those with 4 or more U’s: "UUUGUA" "UUUAUA" "UUUUGU" "UAUUUU" "UUGUAU" "AUUUUU" "UUUUUG" "UUAUAU" "UAUAUU" "CUUUUU" "UUUUUU" "GUUUUU". Figure S7 shows that most U-rich CPEB4 binding sites are in close proximity to one or two downstream A-rich motifs.

We did not include the annotated 3’end of the transcript in Figure S7, because even top CPEB4 binding sites are sometimes located hundreds of nucleotides away from the 3’end. However, as they show high DE count and associate with A-rich 6-mers, we consider them valid binding sites. Whether those represent 3’ends missing from the annotation but are actually expressed in HeLa cells, or have a function as an internal site, is an interesting question, which is beyond the scope of the manuscript.

*Figure 5:
Panel G: Suggest putting in a zoomed-in section to highlight the 'codes' in these transcripts. A quick look in IGV failed to provide a satisfying picture that fits the premise of Fig. 4E for any given transcript and that should be discussed so that we are not left with ambiguous literature again regarding the precise positioning of these elements.*

> Response: For three of the IEG targets, EGR1, FOS and JUNB, we now display in Figure S9 zoomed IGV graphs depicting the sequence around the CPEB4 crosslink sites (blue bars), with the closest A-rich motif underlined by a green bar. In case of FOS, the AATAAA site immediately downstream of the U-rich CPEB4 binding site corresponds to the annotated 3'end of the transcript.

*Figure 7:
Panel B: Given that the effect of CPEB4 stabilisation on protein expression is quite subtle (I couldn't convince myself by looking by eye, except maybe in the control at 4-hours), suggest plotting also the p-EGFR kinetics. There appears to be some variability in the activation between samples (e.g. timepoint 2 in CPEB4 samples seems less than control). A trace of this (or normalisation to it?) may help. FOS is cropped too close to judge and should be cropped so that the full signal is included.*

> Response: We agree with the reviewer that the differences in CPEB4 target protein expression between the timepoints with and without CPEB4 knock-down is marginal. Therefore, we decided to remove this rather unconvincing experiment. One problem could be that we noticed quite a strong variability in the strength of IEG induction between different repeat experiments. In the new Figure 7, we rather focused on the comparative analysis of all four CPEB proteins in terms of their ability to interact with the CCR4-NOT complex and to promote mRNA deadenylation and decay.

The contribution of CPEB4 towards the degradation of IEG mRNAs following mitogenic stimulation with EGF has been analyzed in CPEB4 KO cells and is now part of Figure 6 (see response below).

*Finally, the nail in the coffin of CPEB4 stimulating translation would be showing that the increased protein expression in siRNA samples is due to an increase in mRNA stability by qPCR or 3'-RACE. I think that would be an easy experiment and would be good to add.*

> Response: First, we would like to emphasize that our intention is not to disprove an activating function of CPEB4 in translation control (for which there is ample evidence in the literature). Rather, our manuscript demonstrates that CPEB4 (as well as CPEB1 and CPEB2) is capable of assembling a repressor complex that promotes target mRNA deadenylation and decay. Based on the literature, it is likely that phosphorylation (and maybe other PTMs) is responsible for switching between the activator and repressor functions of CPEB4.

Regarding the role of CPEB4 in EGF-stimulated cells, we added a new set of experiments where we assessed the decay of two CPEB4 target mRNAs, JUNB and FOS. Control and CPEB4 KO cells were subjected to serum deprivation for 24 hours prior to stimulation with EGF for 2 hours, followed by transcriptional shut-off with actD (Figure 6C). Expression analysis showed that both mRNAs are induced 2- to 4-fold upon stimulation with EGF (Figure S12C). In contrast to unstimulated conditions (Figure 6A, Figure S12A), differences in JUNB and FOS mRNA stability were barely detectable between the controls and CPEB4 KO clones upon EGF treatment (Figure 6D, Figure S12D). This is well in line with the observation (as the reviewer pointed out correctly, see comment above) that expression of EGR1 and FOS proteins is very similar between control, CPEB4 KD and rescue cells (former Figure 7B). Hence, it appears that CPEB4 loses its repressor function in EGF-stimulated cells. Accordingly, we changed the interpretation of our results (line 341/342) and thank the reviewer for making this point.

X. laevis CPEB4 has been shown to undergo ERK2-mediated phosphorylation at 7 serine residues within its N-terminal low complexity region, which enhances its ability to promote cytoplasmic polyadenylation (Guillen-Boixet, J., et al., 2016). Indeed, 6 of these sites are conserved in human CPEB4 (Figure S14). Since ERK2 is activated following 2 hours of EGF stimulation (Figure S12E), we speculate that an increase in CPEB4 phosphorylation might trigger the shift towards an activator complex, which would explain that CPEB4 KO has little, or no, effect on IEG mRNA stability in stimulated cells. While CPEB4-mediated mRNA turnover promotes the degradation of IEG mRNAs under basal conditions, ERK2-mediated phosphorylation upon EGF treatment might favor its activity within a cytoplasmic polyadenylation complex (outlined in the discussion on lines 445-451). A detailed analysis of the molecular events that allow switching between the polyadenylation-promoting CPEB4-activator complex and the decay-promoting CPEB4-repressor complex is beyond the scope of our current study and needs to be addressed in future.

**Second round of review**

**Reviewer 2**

I commend the authors on their carefully revised manuscript, their thoughtful responses to my comments and having gone 'over and above' to satisfy both my curiosity as well as my biases for figure layouts and other picky details...
I am very happy to recommend this manuscript for publication and congratulate the team on a thorough body of work that is certain to hold an important place in the field.
I suspect this is just the start of a new level of understanding of how these regulators control spatiotemporal translation of target mRNA. You've piqued my interest about what's going on with cpeb3 and look forward to the next projects that describe what's going on there.
